# Carbon Monoxide Poisoning Resistance and Structural Stability of Single Atom Alloys

**DOI:** 10.1007/s11244-017-0882-1

**Published:** 2018-01-08

**Authors:** Matthew T. Darby, E. Charles H. Sykes, Angelos Michaelides, Michail Stamatakis

**Affiliations:** 1 Thomas Young Centre and Department of Chemical Engineering, University College London, Roberts Building, Torrington Place, London, WC1E 7JE UK; 2 Department of Chemistry, Tufts University, 62 Talbot Ave., Medford, MA 02155 USA; 3 Thomas Young Centre, London Centre for Nanotechnology and Department of Physics and Astronomy, University College London, Gower Street, London, WC1E 6BT UK

**Keywords:** Carbon monoxide, Catalyst stability, Catalyst poisoning, Highly dilute alloy, Platinum group metals, Ensemble effects

## Abstract

Platinum group metals (PGMs) serve as highly active catalysts in a variety of heterogeneous chemical processes. Unfortunately, their high activity is accompanied by a high affinity for CO and thus, PGMs are susceptible to poisoning. Alloying PGMs with metals exhibiting lower affinity to CO could be an effective strategy toward preventing such poisoning. In this work, we use density functional theory to demonstrate this strategy, focusing on highly dilute alloys of PGMs (Pd, Pt, Rh, Ir and Ni) with poison resistant coinage metal hosts (Cu, Ag, Au), such that individual PGM atoms are dispersed at the atomic limit forming single atom alloys (SAAs). We show that compared to the pure metals, CO exhibits lower binding strength on the majority of SAAs studied, and we use kinetic Monte Carlo simulation to obtain relevant temperature programed desorption spectra, which are found to be in good agreement with experiments. Additionally, we consider the effects of CO adsorption on the structure of SAAs. We calculate segregation energies which are indicative of the stability of dopant atoms in the bulk compared to the surface layer, as well as aggregation energies to determine the stability of isolated surface dopant atoms compared to dimer and trimer configurations. Our calculations reveal that CO adsorption induces dopant atom segregation into the surface layer for all SAAs considered here, whereas aggregation and island formation may be promoted or inhibited depending on alloy constitution and CO coverage. This observation suggests the possibility of controlling ensemble effects in novel catalyst architectures through CO-induced aggregation and kinetic trapping.

**Electronic supplementary material:**

The online version of this article (10.1007/s11244-017-0882-1) contains supplementary material, which is available to authorized users.

## Introduction

The platinum group metals (PGMs), including Pd, Pt, Rh and Ir, as well as Ni, are well-known for their excellent activity in a wide variety of heterogeneous catalytic systems; however these metals suffer from CO poisoning as a consequence of their high reactivity [1–3]. In order to prevent CO poisoning, one has to promote the desorption of CO, for instance by operating at elevated temperatures [4]. Not only are high reaction temperatures expensive to employ, but then due to the exothermic nature of adsorption, this would result in low coverages for other important reactants, thereby hampering activity. In addition, the risk of deactivation due to sintering is increased, particularly with supported catalysts. Another strategy that has proven useful for circumventing poisoning (whilst retaining reasonable activity) has been to alloy less reactive metals such as Cu, Ag and Au, with the PGMs; these coinage metals exhibit high tolerance to poisoning, albeit typically having reduced catalytic activity [5–10]. Generally, alloying in this manner quenches the affinity of the PGMs to CO, though it may also inhibit their activity [5–10].

In several cases it has been shown that by doping the coinage metals with PGMs at very low molar fractions, such that these more reactive metals disperse as isolated single atoms in the surface layer of the host material, the activity of the coinage metal surface can be dramatically enhanced whilst retaining excellent reaction selectivity [11–24]. These *single atom alloys* (SAAs) of Sykes and co-workers exhibit tolerance to CO [18] and have been employed to catalyse hydrogenation [16–20], dehydrogenation [22, 23], C–H activation and hydrosilylation [25] reactions with high activity and selectivity, as extended model surfaces and/or as real catalyst nanoparticles.

Temperature programmed desorption (TPD) of CO from Pt/Cu(111) SAA model surfaces revealed that CO desorbs at 350 K from this SAA compared to 450 K from pure Pt(111), indicating weak binding of CO [18]. Under micro-reactor conditions, it was shown that in the presence of 200 ppm CO (a typical industrial concentration in H_2_ streams) the activity of Pt/Cu SAA nanoparticle catalysts for acetylene hydrogenation is reduced twofold, however when compared to monometallic Pt nanoparticles there was a 15-fold activity decrease [18]. It follows that the weak binding of CO to single, isolated Pt atoms in Pt/Cu SAAs compared to that on pure Pt is sufficient to give this material notable resistance to CO poisoning, despite a relatively low number of active sites compared to monometallic Pt [18].

It is with this in mind that we carry out a detailed theoretical study of the effects of CO on an assortment of SAAs comprising single atoms of Ni, Pd, Pt, Rh and Ir doped into Ag(111), Au(111) and Cu(111) surfaces. We perform atomistic calculations using density functional theory (DFT) that are then used to parameterize temperature programmed desorption (TPD) simulations using kinetic Monte Carlo (KMC). Thus, we are able to determine the strength of the interaction of CO and its relation to temperature of desorption from this set of candidate SAAs, with the aim of identifying materials that may exhibit good resistance to CO poisoning.

Additionally, we recognize that the presence of adsorbates may induce structural changes in binary alloy materials, such as segregation of atoms from the bulk into the surface layer, as well as promoting aggregation and island formation [26–34]. Such changes are caused by differences in adsorption behaviour between an adsorbate on each metallic component of the alloy; these differences can offset or increase the energy change upon restructuring of the material. Thus, we perform calculations to determine the segregation and aggregation energies of PGM dopant atoms in highly dilute binary alloys in the absence and presence of CO, allowing us to gauge the stability of SAA materials.

The rest of the paper is organised as follows: we first present the setup of the DFT and KMC calculations in Sect. 2, continuing with Sect. 3 where we explore the interactions of CO with SAAs, in the context of poisoning resistance and adsorbate-induced structural changes. We finally summarise our findings and lessons learned in the “Conclusions” section. Our study should provide a valuable guide for the choice of catalytically active and selective binary alloy combinations that exhibit improved CO tolerance and structural stability.

## Computational Details

### Density Functional Theory Setup

We perform periodic density functional theory calculations using the Vienna ab initio Simulation Package (VASP) version 5.4.1 [35–37] with the projector augmented wave (PAW) method to model core ionic potentials [38, 39] and the revised Perdew–Burke–Ernzerhof (RPBE) exchange–correlation functional [40, 41]. RPBE is chosen in this instance as it was specifically designed to overcome issues of over-binding using other xc-functionals and is proven to give CO adsorption energies that are close to those from experiment [40, 41]. We use a 3 × 3 × 5 slab unit cell whereby we fully relax the top-most four layers while we fix the bottom-most layer at the RPBE bulk FCC lattice constant of the corresponding metal (for SAAs, we use the host material lattice parameters). A vacuum region with thickness of 10 Å separates periodic images in the z-direction. We model exclusively the (111) surface of all materials as this is the surface with the lowest surface free energy for the each host metal in this study [42]. For binary surface alloy calculations, we replace one, two or three surface host atoms with dopant atoms. For calculations where the dopant is in the bulk, we replace a single atom in the 3rd layer of the unit cell with a dopant atom. We use a 13 × 13 × 1 Monkhorst–Pack k-point mesh to sample the Brillouin zone and the planewave kinetic energy cutoff is set to 400 eV. The Methfessel–Paxton smearing width is set to 0.1 eV. We ensure electronic self-consistency up to a tolerance of 10^−7^ eV and during ionic relaxation, we perform minimization of the Hellmann–Feynman forces on free atoms to within a tolerance of 10^−2^ eV Å^−1^. We present adsorption energies ${E_{ads}}(mCO)$, relative to $m$ gas phase CO molecules such that

1 $${E_{ads}}(mCO)=~E_{{Tot}}^{{mCO+slab}} - E_{{Tot}}^{{slab}} - m \cdot E_{{Tot}}^{{C{O_{(g)}}}},$$

where $E_{{Tot}}^{{mCO+slab}}$, $E_{{Tot}}^{{slab}}$ and $E_{{Tot}}^{{C{O_{(g)}}}}$ are the DFT total energies of $m$ CO molecules adsorbed on a slab, the clean slab and gas phase CO, respectively. Thus, negative ${E_{ads}}(mCO)$ means exothermic adsorption. All adsorption configurations of $m$ CO with distinct geometries are given in the supporting information; those with comparable geometries are also noted here.

### Kinetic Monte Carlo Setup

We perform simulations within the graph-theoretical KMC framework as implemented in *Zacros*, version 1.02 [43–45]. We ramp the simulation temperature at a rate of 1 K s^−1^ to simulate TPD. The partial pressure of gas phase CO is set to zero in order to reproduce ultra-high vacuum conditions. The simulation cells consist of (30 × 31) rectangular unit cells with sixfold symmetry. Simulations on SAAs use lattices where host metal sites have been randomly substituted with dopant metal sites giving a final dopant atom percentage density of approximately 1%. We initialize the surface with only dopant sites covered entirely by CO adsorbates (1:1 dopant:CO coverage), since in these materials CO binds significantly more strongly on the dopant, compared to the host sites. We do not account for any CO–CO lateral interactions in TPD simulations on SAA lattices; the high dispersion of single atom sites results in CO adsorbates that reside far from each other and therefore do not interact.

### Rate Constants from Density Functional Theory

In order to perform a KMC simulation, we must first calculate rate constants for CO desorption on each surface. According to transition state theory (TST), the rate constant ${k_{TST}}$ of an elementary process can be calculated as

2 $${k_{TST}}=\frac{{{k_B}T}}{h} \cdot \frac{{{Q^{TS}}}}{{{Q^{IS}}}}\exp \left( { - \frac{{{{{\Delta}}}{E_a}}}{{{k_B}T}}} \right)$$

where $~{k_B}$ is the Boltzmann constant, $h$ is Planck’s constant, $T$ is the temperature, ${Q^{TS}}$ and ${Q^{IS}}$ are the molecular partition functions for the transition state and initial state, respectively, and ${{{\Delta}}}{E_a}$ is the activation barrier. The adsorption of CO is non-activated, so ${{{\Delta}}}{E_a}$ for CO desorption is taken to be ${E_{ads}}(CO)$. Moreover, this implies there is no “explicit” transition state, therefore a 2D gas phase CO transition state is assumed (the third translational degree of freedom is the reaction coordinate: the distance from the surface) [46]. Thus, Eq. (2) for CO desorption becomes

3 $${k_{TST}}=\frac{{{k_B}T}}{h} \cdot \frac{{{Q^{C{O_{(g)}}}}}}{{{Q^{C{O^*}}}}} \cdot \exp \left( {\frac{{{E_{ads}}}}{{{k_B}T}}} \right).$$

We compute the partition functions $Q$ using the vibrational frequency data in Table 1, under the harmonic approximation [46]. The pre-exponential factor in (3) is temperature dependent, both due to the thermal factor of ${k_B}T{\text{/}}h$, but also because ${Q^{C{O_{(g)}}}}$ and ${Q^{C{O^*}}}$ are functions of $T$ [46]; this is accounted for in the KMC simulation using fitted functions of $T$.

**Table 1:** Adsorption energies (E_ads_(CO)) and vibrational frequencies ($\nu$) for CO chemisorption at the most favourable adsorption site on pure metal and SAA (111) surfaces

Surface	Site	E_ads_ (CO) (eV)	Vibrational frequencies (cm^−1^)					
			${\nu _1}$	${\nu _2}$	${\nu _3}$	${\nu _4}$	${\nu _5}$	${\nu _6}$
Ag(111)	Top	0.02	2015	144	121	120	11	20i
Au(111)	Top	− 0.05	2035	257	169	168	33	3i
Cu(111)	Fcc	− 0.51	1800	263	224	223	113	110
Ni(111)	Fcc	− 1.50	1749	331	267	266	138	134
Pd(111)	Fcc	− 1.67	2011	394	296	295	32	28
Pt(111)	Fcc	− 1.48	1728	329	301	300	155	153
Rh(111)	Hcp	− 1.62	1726	329	274	274	147	147
Ir(111)	Top	− 1.83	1998	497	446	446	67	63
Ni/Ag(111)	Top	− 1.57	1946	412	334	333	53	45
Ni/Au(111)	Top	− 1.31	1991	395	336	336	51	48
Ni/Cu(111)	Top	− 1.39	1972	407	342	342	49	44
Pd/Ag(111)	Top	− 0.98	1982	367	261	260	45	36
Pd/Au(111)	Top	− 0.88	2018	353	268	268	41	37
Pd/Cu(111)	Top	− 0.84	2007	357	277	276	33	23
Pt/Ag(111)	Top	− 1.41	1977	449	334	333	52	46
Pt/Au(111)	Top	− 1.37	2014	450	352	352	53	51
Pt/Cu(111)	Top	− 1.18	2001	439	345	345	48	47
Rh/Ag(111)	Top	− 1.98	1939	446	358	357	51	46
Rh/Au(111)	Top	− 1.78	1977	440	375	375	57	48
Rh/Cu(111)	Top	− 1.71	1972	432	371	370	49	42
Ir/Ag(111)	Top	− 2.47	1938	507	413	412	57	51
Ir/Au(111)	Top	− 2.31	1977	503	429	429	63	59
Ir/Cu(111)	Top	− 2.09	1970	488	418	417	54	50

The vibrational modes can be described as follows; ${\nu _1}$ C–O stretch, ${\nu _2}$ M–C stretch, ${\nu _{3/4}}$ hindered rotations and ${\nu _{5/6}}$ hindered translations. The imaginary frequencies on Ag and Au can be attributed to numerical artefacts in the calculations of the soft translational modes

## Results and Discussion

### CO Adsorption on Pure Metal and SAA Surfaces

**Fig. 1:**
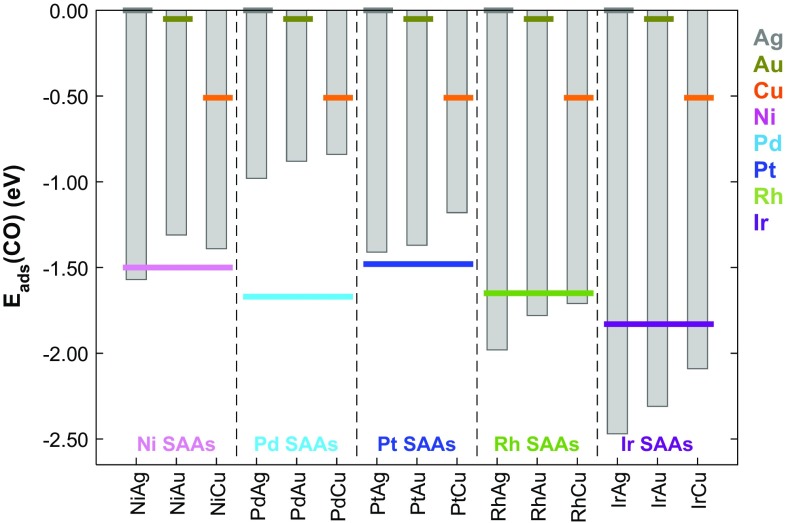
Adsorption energies (E_ads_) of CO on the most stable sites of SAA (111) surfaces. Corresponding values of E_ads_ for pure metal (111) surfaces are shown by coloured horizontal lines [Ag (grey), Au (gold), Cu (orange), Ni (pink), Pd (cyan), Pt (blue), Rh (green) and Ir (purple)]

Using DFT with the RPBE xc-functional, we calculate the geometry of a CO molecule chemisorbed on pure metal and SAA (111) surfaces. For the pure metal (111) surfaces, our calculations are in excellent agreement with the works of others [47, 48]. We determine that the CO interactions with Ag(111), Au(111) and Cu(111) are much weaker than for Ni(111), Pd(111), Pt(111), Rh(111) and Ir(111) (Fig. 1; Table 1).

For CO adsorption on a top site on Ag(111), we calculate the value of ${E_{ads}}(CO)$ to be 0.02 eV indicating a slightly endothermic binding with the functional used, whereas on the top site of Au(111) there is a marginally exothermic CO adsorption energy ${E_{ads}}$(CO) of − 0.05 eV. These values are in agreement with experimental observations that CO binds weakly to these surfaces only at low temperatures [49–51]. CO adsorption on Cu(111) is exothermic and is most favourable in fcc hollow sites though much stronger than on Au(111), with an adsorption energy of − 0.51 eV with the functional used. For Ni(111), Pd(111) and Pt(111), the most stable site for adsorption is also the fcc hollow site with adsorption energies of − 1.50, − 1.67 and − 1.48 eV. CO adsorption on Rh(111) is most favourable on the hcp hollow site with an adsorption energy of − 1.65 eV. Finally, for Ir(111) the most stable adsorption site is the top site with the largest pure metal adsorption energy of − 1.83 eV.

Though our predictions of both the adsorption energy and preferred adsorption site for CO agree well with the theoretical work of others, it should be noted that the prediction of adsorption site preference by DFT under the generalized gradient approximation is qualitatively incorrect; several explanations and remedies for this phenomenon have been reported, with an excellent discussion by Kresse et al. suggesting this is due to an overestimation of the HOMO–LUMO gap in CO [52, 53]. Low temperature experiments reveal that CO prefers to bind on one-fold rather than three-fold adsorption sites on Cu(111) and Pt(111), in disagreement with DFT.

We determine that on each of the SAAs considered here, the most favoured adsorption site for CO is the top site of the single dopant atom; geometry optimizations starting with CO on shared bridge or hollow sites of SAAs typically result in CO being displaced back to the dopant top site. We report adsorption energies of CO in the most favourable adsorption sites on SAA (111) surfaces in Fig. 1 and Table 1. The calculations on Pt/Cu(111) and Ni/Cu(111) are in good agreement with previous works on the adsorption of CO on Ni and Pt impurity atoms at ¼ ML coverage in Cu(111) [54].

To quantify the relative change in CO adsorption strength on SAAs $E_{{ads}}^{{SAA}}(CO)$ relative to its monometallic host $E_{{ads}}^{{host}}(CO)$ and dopant $E_{{ads}}^{{dopant}}(CO)$, we use the following equation

4 $$\varphi (CO)=~\frac{{E_{{ads}}^{{dopant}}(CO) - E_{{ads}}^{{SAA}}(CO)}}{{E_{{ads}}^{{dopant}}(CO) - E_{{ads}}^{{host}}(CO)}}.$$

Values of $~0<\varphi (CO)<1$ indicate that CO adsorption on these SAAs is weaker than on pure dopant surfaces but stronger than on pure host materials, whereas values where $\varphi (CO)<0$ indicate CO adsorption strength that is greater than on the monometallic dopant. We see for all Pd- and Pt-doped materials, as well as Ni/Au(111) and Ni/Cu(111) that $0<\varphi (CO)<1$, therefore CO adsorption on these SAAs is weaker than on pure dopant surfaces (Ni, Pd, Pt) though stronger than on pure host surfaces (Ag, Au, Cu). The most notable reductions in CO adsorption strength compared to the pure dopant materials are on Pd SAAs with $\varphi (CO)$ calculated to be 0.40, 0.49 and 0.72 for Pd/Ag(111), Pd/Au(111) and Pd/Cu(111) respectively. For Pt SAAs, there is still a significant $\varphi (CO)$ for Pt/Cu(111) of 0.31, though only smaller values of 0.05 and 0.08 for Pt/Ag(111) and Pt/Au(111), respectively. Ni/Au(111) and Ni/Cu(111) have $\varphi (CO)$ values of 0.13 and 0.12, respectively. However, Ni/Ag(111) as well as all Rh- and Ir-doped SAAs have values of $\varphi (CO)<0$. CO binds more strongly to these SAAs and thus these materials will not offer any resistance to CO poisoning. However, if we use the adsorption energy of CO as a gauge of reactivity, these Rh- and Ir-doped SAAs may be useful for other applications, such as CO dissociation catalysts.

### Temperature Programmed Desorption Simulations

Reductions in the adsorption strength of CO on SAAs compared to pure dopant surfaces will result in an increased tolerance to catalytic poisoning by CO. We quantify this resistance to poisoning by simulating CO TPDs from (111) surfaces of metals and alloys of interest using KMC and comparing desorption peak temperatures. To evaluate the quality of our dataset, we compare these peak temperatures to experimental ones for the pure metals (excluding Ag and Au, due to weak or no binding) and several SAAs that have been synthesized experimentally (Ni/Cu(111), Pd/Au(111), Pd/Cu(111) and Pt/Cu(111)).

### Simulated Desorption Peak Temperatures

**Fig. 2:**
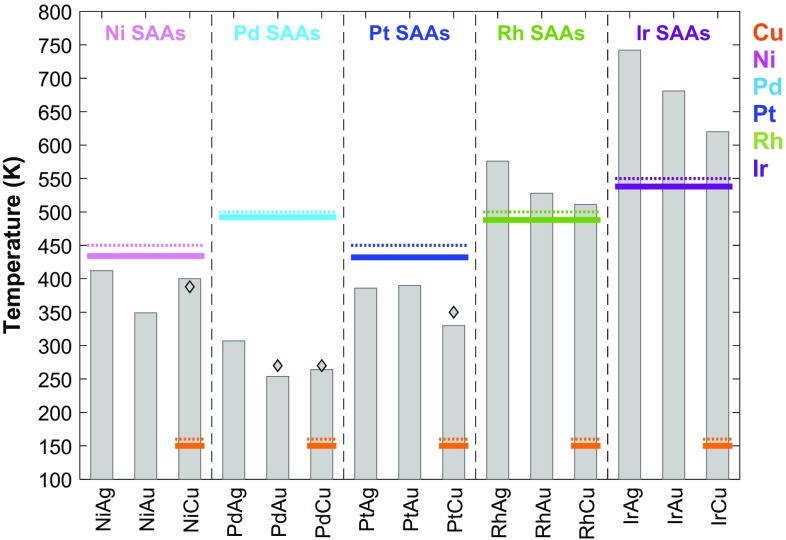
Peak desorption temperatures from KMC simulated TPD of CO on SAA (111) surfaces. Temperatures from pure metal simulations are shown as full-horizontal lines whereas corresponding temperatures from experiment [55–58] are shown as dotted-horizontal lines [Cu (orange), Ni (pink), Pd (cyan), Pt (blue), Rh (green) and Ir (purple); Au and Ag not shown due to weak or no CO binding]. Experimental SAA temperatures are shown with diamonds [16, 18, 21, 55]

We now examine the thermal desorption of CO on each pure metal and SAA (111) surface. During a TPD simulation, we record the coverage of CO* (Θ_CO_) on the lattice, as well as the number of gas molecules evolved from the surface, at intervals of 0.25 s. The TPD signal is obtained as a moving average of the instantaneous desorption rate, thereby allowing us to determine the time and temperature (1 K s^−1^ ramp rate) that the rate of CO desorption is greatest. The corresponding peak desorption temperature, T_sim_, is plotted for all surfaces in Fig. 2, alongside any known experimental data [16, 18, 21, 55–58]. Comparing our simulated TPD peak temperatures to this data, we can see that there is excellent agreement with a mean absolute error of 13 K, providing good support for the reliability of our model and dataset.

The majority of SAAs show reductions in CO desorption temperature over their monometallic dopant analogues, including all Ni-, Pd- and Pt-doped SAAs. In particular, we see that there is over a 220 K decrease in the desorption temperature of Pd/Au(111) and Pd/Cu(111) SAAs compared to monometallic Pd, as well as a 185 K decrease with Pd/Ag(111). For Pt doped SAAs, the largest peak temperature reduction of 102 K is simulated for Pt/Cu(111), whereas Pt/Ag(111) and Pt/Au(111) exhibit desorption temperature reductions of over 40 K compared to pure Pt(111). Despite a higher adsorption energy on Ni/Ag(111) compared to pure Ni(111), CO desorbs with a peak temperature that is 22 K lower from this SAA. This qualitative deviation from the expected relationship between adsorption energy and TPD peak temperature is attributed to the soft vibrational modes of CO bound to Ni/Ag(111) (Table 1, ${\nu _5}$ and ${\nu _6}$) giving a lower desorption pre-exponential for this material. This difference offsets the stronger adsorption of CO on Ni/Ag(111) [0.07 eV difference versus Ni(111)]. We also simulate TPD peak temperatures for Ni/Au(111) and Ni/Cu(111) that are 85 and 34 K below that of Ni(111), respectively.

For Ir- and Rh-doped SAAs, we simulated CO TPD peak temperatures that are above the corresponding temperatures for desorption from pure Rh(111) and Ir(111), in line with stronger adsorption of CO on these SAAs. We calculate 88, 40 and 23 K increases in the TPD peak temperatures for CO desorbing from Rh/Ag(111), Rh/Au(111) and Rh/Cu(111), respectively. For Ir-doped SAAs, the analogous temperature differences are greater, being 204, 143 and 82 K for Ir/Ag(111), Ir/Au(111) and Ir/Cu(111), respectively.

**Fig. 3:**
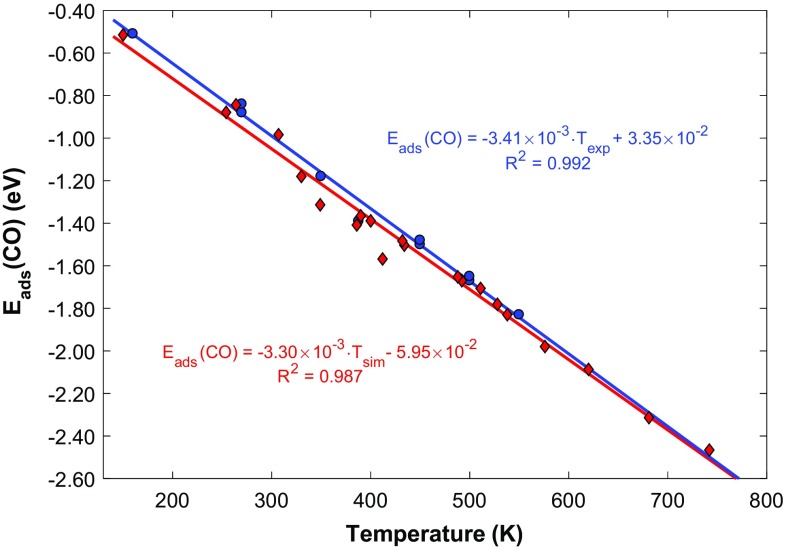
Linear correlation of TPD peak temperatures from experiment (blue) and KMC simulation (red) with the adsorption energy of CO calculated from DFT. Regression equations are shown with corresponding coefficients of determination (R^2^). Standard errors in the slope and intercept for the experimental case are ± 1.11 × 10^−4^ and ± 4.51 × 10^−2^, respectively. The analogous standard errors in the slope and intercept for the KMC simulated case are ± 8.79 × 10^−5^ and ± 4.08 × 10^−2^, respectively

Our data suggests that there exists a strong linear correlation between E_ads_(CO) and the TPD peak temperature (both for T_exp_ and T_sim_) (Fig. 3). This finding is typical for a first order desorption process as predicted by an equation first derived by Redhead [59]. Though useful, the Redhead equation can often produce errors as a result of poor estimation of the pre-exponential; for example, arbitrarily choosing a pre-exponential value of k_B_T_exp_/h (as is typical) gives a mean absolute error in the Redhead value of E_ads_(CO) of 0.20 eV compared to the DFT adsorption energy. In our case, we have calculated temperature dependent pre-exponentials using TST, assuming harmonic vibrational modes. This may not always be an accurate approximation, especially when considering very soft, frustrated translations or rotations in the partition function [60], though good agreement with experiment supports the use of harmonic TST in this case. We pose that our calibrated linear fitting of

5 $${E_{ads}}(CO)=~ - 3.30 \times {10^{ - 3}} \cdot {T_{sim}} - 5.95 \times {10^{ - 2}}$$

may be used for quick extraction of the CO adsorption energy from future experimental work. This fitting is specific for DFT using the RPBE xc-functional, though good agreement of our data with experiment supports the choice of this xc-functional in this case. We calculate the mean absolute error in the fitting to be 0.04 eV.

Our DFT results show that highly diluted alloys of single atoms, out of the catalytically active group 10 metals, dispersed into more inert hosts, out of group 11 metals, can reduce the adsorption strength of CO, compared to the pure dopants. In line with these results, KMC simulations reveal that the CO desorption temperature may be reduced by more than 220 K in some cases, thereby dramatically reducing the susceptibility of the surface to poisoning by CO. Decreased TPD peak temperatures imply that it is not necessary to heat these SAA catalytic systems to temperatures as high as on pure dopant surfaces in order to circumvent CO poisoning. Thus, one can carry out the reaction on SAAs at lower temperatures, thereby reducing the risk of catalyst sintering and hampered reaction selectivities. However, increases in the CO desorption temperature for Rh- and Ir-doped SAAs indicate reduced tolerance to CO over their monometallic constituents and an increased susceptibility to CO poisoning.

Our study so far has assumed that under conditions where CO is present, the SAA structure is indeed favourable. However it is well known that adsorbates, in particular those that are as potent as CO, may induce changes in the surface structure of a material through effects such as segregation or formation of islands (clusters) on the surface. It is with this in mind that we move on to study the stability of the SAA structure with respect to the aforementioned phenomena, both in the absence and presence of CO. This stability analysis will serve as a guide for the experimental synthesis of SAAs, highlighting those metal combinations with an enthalpic preference for the SAA structure over other phases, still consistent with high dopant dilution.

### Adsorbate-Induced Structural Changes in SAAs

Under realistic conditions, restructuring of the surface of a catalyst can result in modifications to its function. The low concentration of dopant atoms in a SAA means that effects such as dopant atom segregation into the bulk or clustering on the surface, would result in a fundamental change in the surface structure of the material and transformation of a SAA into some other class of binary alloy. In particular, segregation of a single dopant atom into the bulk may result in decreased catalytic activity that more closely resembles the host material. Moreover, clustering of dopant atoms in the surface layer may result in dimer, trimer and even island formation which will hamper the selectivity and poisoning resistance of the surface.

Thus, in this section we investigate the thermodynamic stability of SAAs under vacuum conditions and also in the inevitable presence of CO under operating conditions. We use DFT to calculate energy changes between the SAA structure and other highly dilute analogues where the SAA is buried into the bulk structure of the host or aggregated into clusters on the surface. By comparing the values of these energy changes in the absence versus presence of CO, we quantify the effect of this species on SAA stability.

### Surface Segregation

We perform calculations for each binary alloy in structures where a single dopant atom is in the surface layer (i.e. a SAA) or immersed in the bulk material. For the latter, we approximate the “bulk” as a single dopant atom in the 3rd layer of the host material slab such that the dopant atom is fully coordinated to host atoms and the slab is symmetric. The segregation energy $\Delta {E_{seg}}$ is then computed relative to the SAA phase such that

6 $$\Delta {E_{seg}}=~{E_{Tot}}(bulk) - {E_{Tot}}(SAA);$$

where ${E_{Tot}}(bulk)$ and ${E_{Tot}}(SAA)$ are the DFT total energies of the single dopant atom immersed in the 3rd layer of the host material slab and the single dopant atom in the surface layer of the host slab respectively. Note that positive values of $\Delta {E_{seg}}$ correspond to a preference for segregation of the dopant to the surface, not accounting for entropy.

**Fig. 4:**
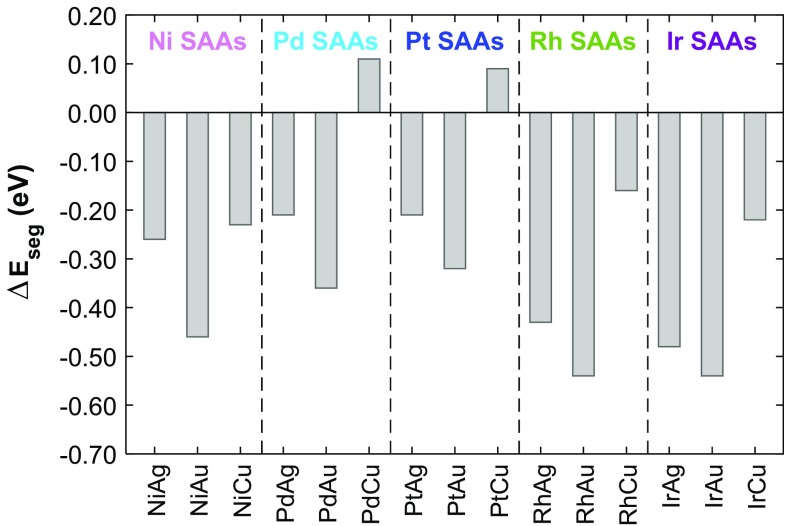
The segregation energy $\Delta {E_{seg}}$ for a single dopant atom to migrate from the (111) surface of a group 11 host material into the “bulk”. Positive values indicate a preference for segregation of the dopant to the surface, whereas negative values into the bulk

Our calculations show that for most metal combinations considered here, it is more favourable for a single dopant atom to reside in the bulk rather than at the surface (Fig. 4). The only exceptions are Pd/Cu(111) and Pt/Cu(111) whose $\Delta {E_{seg}}$ values are 0.11 and 0.09 eV, respectively. These results show good qualitative agreement with the work of Ruban et al. in a previous study on the surface segregation of transition metal impurity atoms in close-packed transition metal hosts [61].

### CO Induced Surface Segregation

In the presence of CO, our calculations suggest that it is strongly favoured for a single dopant atom to segregate to the surface. To deduce an expression for the segregation energy, we consider a cyclic process entailing: (i) desorption of CO from an alloy structure with the dopant in the bulk, (ii) segregation of the dopant in the surface, (iii) adsorption of CO in the dopant site of the SAA, and (iv) migration of the dopant back to the bulk in the presence of CO, such that the final state is identical to the initial configuration (see supporting information). The CO induced segregation energy $\Delta E_{{seg}}^{{CO}}$ is therefore given as:

7 $$\Delta E_{{seg}}^{{CO}}=\Delta {E_{seg}}+\left\{ {E_{{ads}}^{{host}}(CO) - E_{{ads}}^{{SAA}}(CO)} \right\}$$

**Fig. 5:**
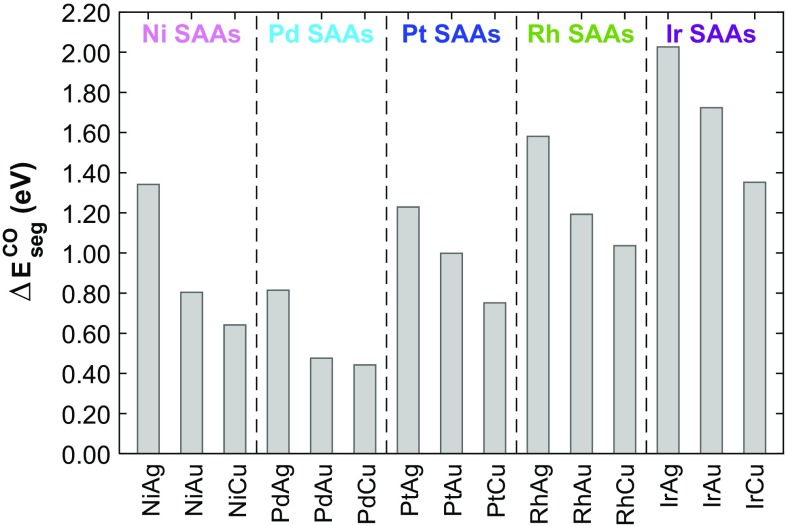
The CO induced segregation energy $\Delta E_{{seg}}^{{CO}}$ for a single dopant atom to migrate from the (111) surface into the “bulk” of group 11 metal host materials. Positive values indicate a preference for segregation of the dopant in the presence of CO to the surface, whereas negative values would indicate a preference for segregation into the bulk

The adsorption strength of CO to SAAs $E_{{ads}}^{{SAA}}(CO)$ is sufficiently greater than that on the pure host materials $E_{{ads}}^{{host}}(CO)$, particularly when considering Ag and Au, such that $\Delta E_{{seg}}^{{CO}}$ is positive in all cases (Fig. 5). Hence, the presence of CO is expected to induce the segregation of the dopant atom to the surface. This has previously been noted experimentally for dilute Pd/Cu SAA nanoparticles whereby exposure to CO pulls Pd to the surface and consequently enhances the activity of these nanoparticles towards acetylene hydrogenation [26]. Moreover, several theoretical studies have demonstrated the phenomenon of adsorbate induced segregation [27–33]. For example a study by Sansa et al. on the CO induced segregation of single transition metal dopant atoms in Au reveals that the adsorption energy of CO is sufficient to promote dopant atom segregation to both the Au(111) and Au(100) surfaces from the bulk [32]. This study by Sansa et al. predicts over-bound CO adsorption energies due to the use of the traditional PBE exchange–correlation functional rather than RPBE used in this case; though their values of $\Delta E_{{seg}}^{{CO}}$ are still in excellent agreement with ours due to a cancellation of the over-binding when the difference is taken between CO bound on a host and SAA material [32].

The CO induced segregation energy is sufficient to ensure dopant atoms will not segregate into the bulk material in the presence of CO. The configurational entropy of a single atom in the bulk is greater than that of the surface due to the high number of bulk sites relative to surface sites. This provides a driving force for dopant atom segregation into the bulk from the surface, though we believe this is likely to be relevant only in cases when CO is not present. However, we hypothesize that diffusion barriers of the dopant moving into the bulk will be sufficiently high to kinetically trap the dopant in the surface layer; this point is particularly pertinent due to the methods of synthesis (vapour deposition) for extended SAA surfaces and SAA nanoparticles (galvanic replacement) involving addition of the dopant atoms directly into the surface layer. This hypothesis is evidenced to some extent thanks to the experimental synthesis of Ni/Au, Ni/Cu, Pd/Au and Pt/Au SAAs, all of which have negative values of $\Delta {E_{seg}}$.

### Surface Aggregation and Island Formation

To evaluate the stability of single isolated dopant atoms towards aggregation, we vary the molar fraction of CO in the surface layer and compute the DFT energies for dimer and trimer configurations on the surface. The aggregation energy for a cluster of n atoms $\Delta {E_{agg}}(n)$ is given relative to a SAA such that

8 $$\Delta {E_{agg}}(n)=~{E_{Tot}}(n)+(n - 1) \cdot {E_{Tot}}(host) - n \cdot {E_{Tot}}(SAA);$$

where ${E_{Tot}}(n)$ and ${E_{Tot}}(host)$ are the DFT total energies of an alloy surface with a cluster of n dopant atoms and the pure host material, respectively. In this case, values of $\Delta {E_{agg}}(n)$ that are negative correspond to a preference for surface clustering, whereas positive values correspond to a preference for dopant atom dispersion to the SAA structure.

**Fig. 6:**
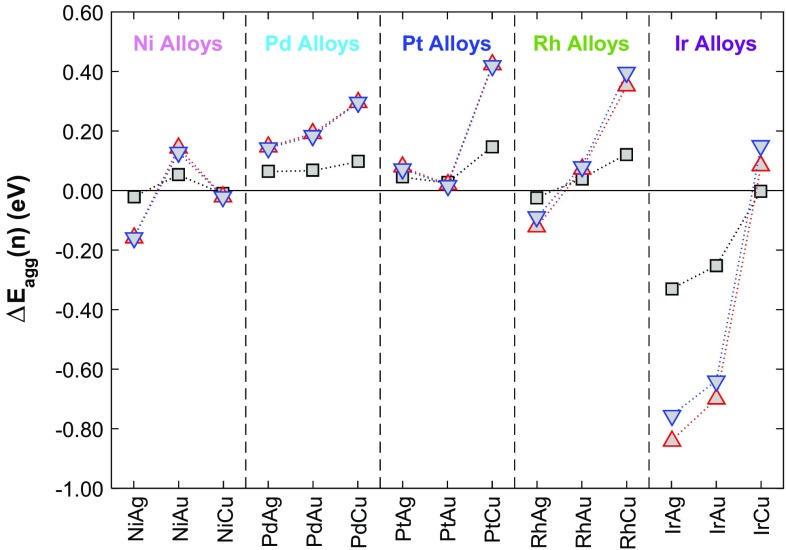
The energy of aggregation $\Delta {E_{agg}}(n)$ relative to the SAA phase for the clustering of group 10 dopant atoms in the (111) surface of group 11 metals into dimers (black squares) and trimers where dopant atoms surround fcc (red up-triangles) and hcp (blue, down-triangles) sites

For the majority of metal combinations we consider here, our calculations show that single dopant atom isolation is favoured for unit cell concentrations up to 1/3 ML, with the exception of Ni-, Ir- and Rh- doped Ag(111), Ir/Au(111) and Ni/Cu(111) which have negative $\Delta {E_{agg}}(n)$ values (Fig. 6). In the case of Ni/Cu(111), the values of $\Delta {E_{agg}}(2)$ and $\Delta {E_{agg}}(3)$ are so small that at temperatures likely to be used in experimental practice, there will be a sufficient entropic tendency to drive surface dopant atoms apart. In fact, configurational contributions to the entropy term, which we do not explicitly consider here, will always favour the SAA structure over aggregation due to the greater disorder of having several, isolated atoms over having a cluster. We can therefore conclude that any system with an enthalpic preference at 0 K for the SAA phase over the aggregated phase will also be more thermodynamically stable as a SAA (i.e. Ni- and Rh-doped Au(111), as well as Ir- and Rh-doped Cu(111) and all the Pd- or Pt-doped materials).

### CO Induced Aggregation and Island Formation

To determine whether exposure of the surfaces to CO may induce aggregation, we compute the adsorption energies of 1, 2 and 3 CO molecules adsorbed to dimer and trimer dopant aggregates.

**Fig. 7:**
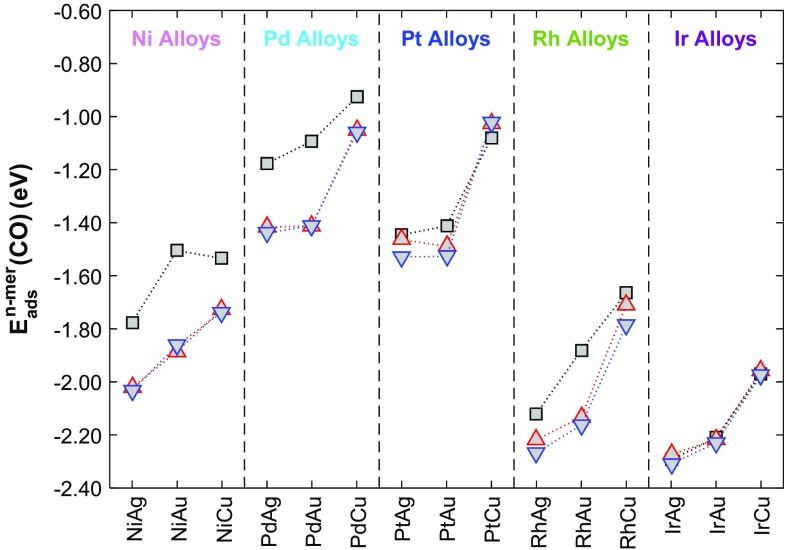
Adsorption energies of a single CO molecule in the most energetically favourable surface site for dimers (black squares) and triangular trimers surrounding fcc (red up-triangles) and hcp (blue down-triangles) of binary alloy combinations of Ni, Pd, Pt, Rh and Ir doped into group 11 (111) surfaces

By performing calculations with just a single CO molecule adsorbed to binary alloy surfaces, we are effectively considering the case where the CO partial pressure is sufficiently low that full saturation of dopant atoms in dimer and trimer configurations is not possible. At this CO coverage, we determine that the CO adsorption energy on an n-mer $E_{{ads}}^{{n{\text{-}}mer}}(CO)$ is greater than the CO adsorption energy on the corresponding SAA $E_{{ads}}^{{SAA}}(CO)$ for all Ni-doped and Pd-doped alloys, as well as Pt- and Rh-doped Ag(111) and Au(111) based SAAs (Fig. 7). The most stable adsorption sites for a single CO molecule on these binary surfaces are the bridge sites connecting two adjacent dopant atoms in dimers, and the hollow sites surrounded by three dopant atoms in triangular trimers. Exceptions to this are Pt/Cu(111), Ir/Ag(111), Ir/Au(111) and Ir/Cu(111) for which it is more favourable for one CO molecule to adsorb on the top site of the SAA, dimer or trimer analogues rather than on two- or three-fold sites.

**Fig. 8:**
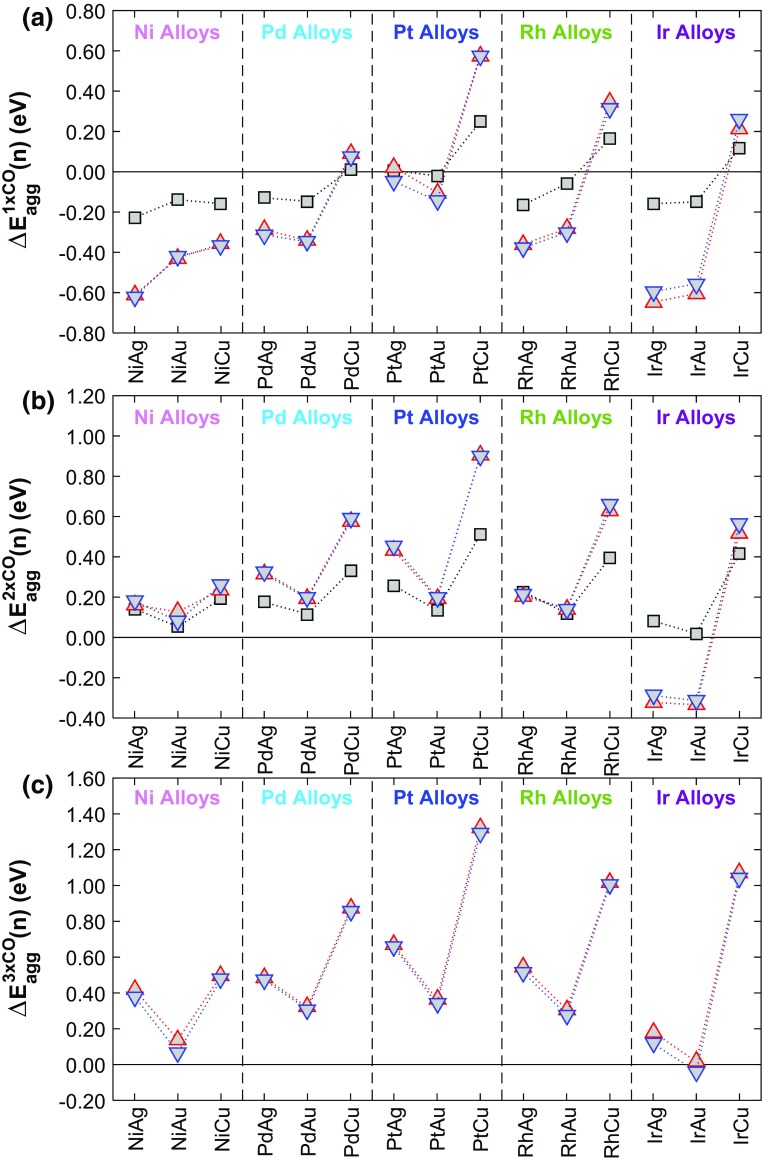
CO induced aggregation energies $\Delta E_{{agg}}^{{m \times CO}}(n)$ for clusters of n atoms relative to the SAA phase of Ni, Pd, Pt, Rh and Ir doped into the (111) surfaces of the group 11 metals with **a** 1 adsorbed CO molecule; **b** 2 adsorbed CO molecules; and **c** 3 adsorbed CO molecules

In order for a single CO molecule to induce aggregation, the adsorption energy of CO on an n-mer island $E_{{ads}}^{{n{\text{-}}mer}}(CO)$ must be more negative than that on a SAA and the difference must also offset positive values of $\Delta {E_{agg}}(n)$ to make the CO induced aggregation energy $\Delta E_{{agg}}^{{m \times CO}}(n)$ negative (Fig. 8a);

9 $$\Delta E_{{agg}}^{{m \times CO}}(n)=~\Delta {E_{agg}}(n) - \left\{ {m \cdot E_{{ads}}^{{SAA}}(CO) - E_{{ads}}^{{n{\text{-}}mer}}(mCO)} \right\}$$

For binary alloys of Pd, Pt, Ir and Rh doped into Cu(111), $\Delta E_{{agg}}^{{1 \times CO}}(n)$ is always positive indicating that these metal combinations will have an energetic preference for dispersion into the SAA phase when exposed to low CO partial pressures. Pt/Ag(111) has values of $\Delta E_{{agg}}^{{1 \times CO}}(n)$ in dimers and trimers that are very close to 0 eV, indicating little or no preference for the SAA phase over other aggregated phases. All other alloys including Ni/Au(111), Pd/Au(111), Pt/Au(111), Rh/Au(111) and Pd/Ag(111) whose values of $\Delta {E_{agg}}(n)$ were all positive (favouring dispersion in the absence of CO) have $\Delta E_{{agg}}^{{1 \times CO}}(n)$ values that are negative, indicating that CO will induce aggregation in these cases.

Under reaction conditions, it is most likely that there will be sufficient CO present to have at least a 1:1 dopant:CO ratio and so we go on to investigate if multiple CO adsorbates may promote aggregation (Fig. 8b, c). The adsorption energy of* m* CO molecules ($m>1$) on* m* top sites of a cluster of* n* dopant atoms $E_{{ads}}^{{n{\text{-}}mer}}(mCO)$ is always notably more negative than that of a single CO molecule in its most favoured adsorption site. We also find that $E_{{ads}}^{{n{\text{-}}mer}}(mCO)$ is more positive than the sum of $m \cdot E_{{ads}}^{{SAA}}(CO)$ for all alloy combinations. In these cases, the CO geometries are tilted away from one another and no longer in line with surface normal as is the case with one CO molecule, indicting the presence of repulsive lateral interactions. The lateral interactions appear to be approximately pairwise additive on trimer clusters.

It follows on that $\Delta E_{{agg}}^{{m \times CO}}(n)$ is made more positive by the presence of multiple CO for the highly dilute binary surfaces we consider here. In fact, negative values of $\Delta {E_{agg}}(n)$ are offset when 2 or 3 CO molecules are co-adsorbed to clustered islands such that $\Delta E_{{agg}}^{{m \times CO}}(n)$ is positive for all cluster sizes for the majority of alloy combinations. Thus, for partial pressures of CO giving fractional coverages of CO on dopant atoms of 0.5–1, dispersion of dopant atoms into the SAA phase will be favoured for these alloys, rather than aggregation into clustered islands. The only SAAs that do not adhere to this statement are Ir/Ag(111) and Ir/Au(111); in these cases each additional CO molecule adsorbed to the surface reduces the negativity of $\Delta E_{{agg}}^{{m \times CO}}(n)$ through repulsive interactions between CO molecules, until 3 CO molecules on trimer configurations or 2 CO molecules on dimer configurations is sufficient to make $\Delta E_{{agg}}^{{m \times CO}}(n)$ positive.

DFT results in the absence of CO, show that most surface alloys tend to be dispersed forming SAAs at high dopant atom dilution. Notable exceptions (at least from an energetic point of view) are Ni-, Ir- and Rh-doped Ag(111), as well as Ni/Cu(111) and Ir/Au(111). Adsorption of a single CO molecule changes this picture for all Au-based SAAs as well as for Ir- and Pd-doped Ag(111) as in the presence of relatively low amounts of CO on the surface, the formation of dimers/trimers may be favoured. On the other hand, for Pd, Pt, Ir and Rh doped into Cu(111) in addition to Pt/Ag(111) dispersion of dopant atoms is favourable despite the presence of CO. For high CO coverages, the repulsive CO–CO lateral interactions are expected to promote the dispersion of dopant atoms, yielding SAA structures for all binary metals considered here. These findings may present an interesting opportunity of controlling ensemble effects, by engineering novel materials with primarily dimers or trimers on the surface of these materials through manipulation of CO partial pressures [29, 62].

## Conclusion

In this study, we have investigated the CO adsorption properties on highly dilute binary alloys of the platinum group metals doped into group 11 metal hosts, with focus on the fcc(111) surface. Using a combination of DFT with KMC, we have shown that Ni-, Pd- and Pt-doped SAAs offer resistance to catalytic CO poisoning as evidenced by reduced CO adsorption energies and CO peak desorption temperatures as compared to pure Ni(111), Pd(111) and Pt(111). On the other hand Rh- and Ir-doped SAAs bind CO more strongly than pure Rh(111) and Ir(111), indicating these SAAs may offer enhanced reactivity over their monometallic counterparts, though also a lack of CO tolerance. Additionally, we have evaluated the stability of SAAs compared to other binary alloy structures and determined that the formation of dispersed structures (i.e. the SAA phase) is energetically favourable in a number of cases. We have considered the effect of CO on these alloys and determined that CO favours the segregation of single dopant atoms into the surface layer of the host material. Moreover, at CO dopant fractional coverage of > 0.5, our calculations suggest that CO will promote dopant atom dispersion in the surface layer, whereas lower CO coverages may favour aggregation leading to the formation of dimers or trimers.

## Electronic supplementary material

Below is the link to the electronic supplementary material.

Supplementary material 1 (DOCX 38483 KB)
