# Supplementary material for: Carbon Monoxide Poisoning Resistance and Structural Stability of Single Atom Alloys
Source: Top Catal. 2018 Jan 8;61(5):428–38. doi: 10.1007/s11244-017-0882-1 (PMC6560695; doi:10.1007/s11244-017-0882-1)
Supplement: Supplementary file 1 — Supplementary material 1 (DOCX 38483 KB) [file 11244_2017_882_MOESM1_ESM.docx]

Supporting Information:

Carbon Monoxide Poisoning Resistance and Structural Stability of Single Atom Alloys

*Matthew T. Darby^a^, E. Charles. H. Sykes^b^, Angelos Michaelides^c^ and Michail Stamatakis^a^*

*a Thomas Young Centre and Department of Chemical Engineering, University College London, Roberts Building, Torrington Place, London, WC1E 7JE, United Kingdom*

*b Department of Chemistry, Tufts University, 62 Talbot Ave., Medford, Massachusetts 02155, United States.*

*c Thomas Young Centre, London Centre for Nanotechnology and Department of Physics and Astronomy, University College London, Gower Street, London WC1E 6BT, United Kingdom*


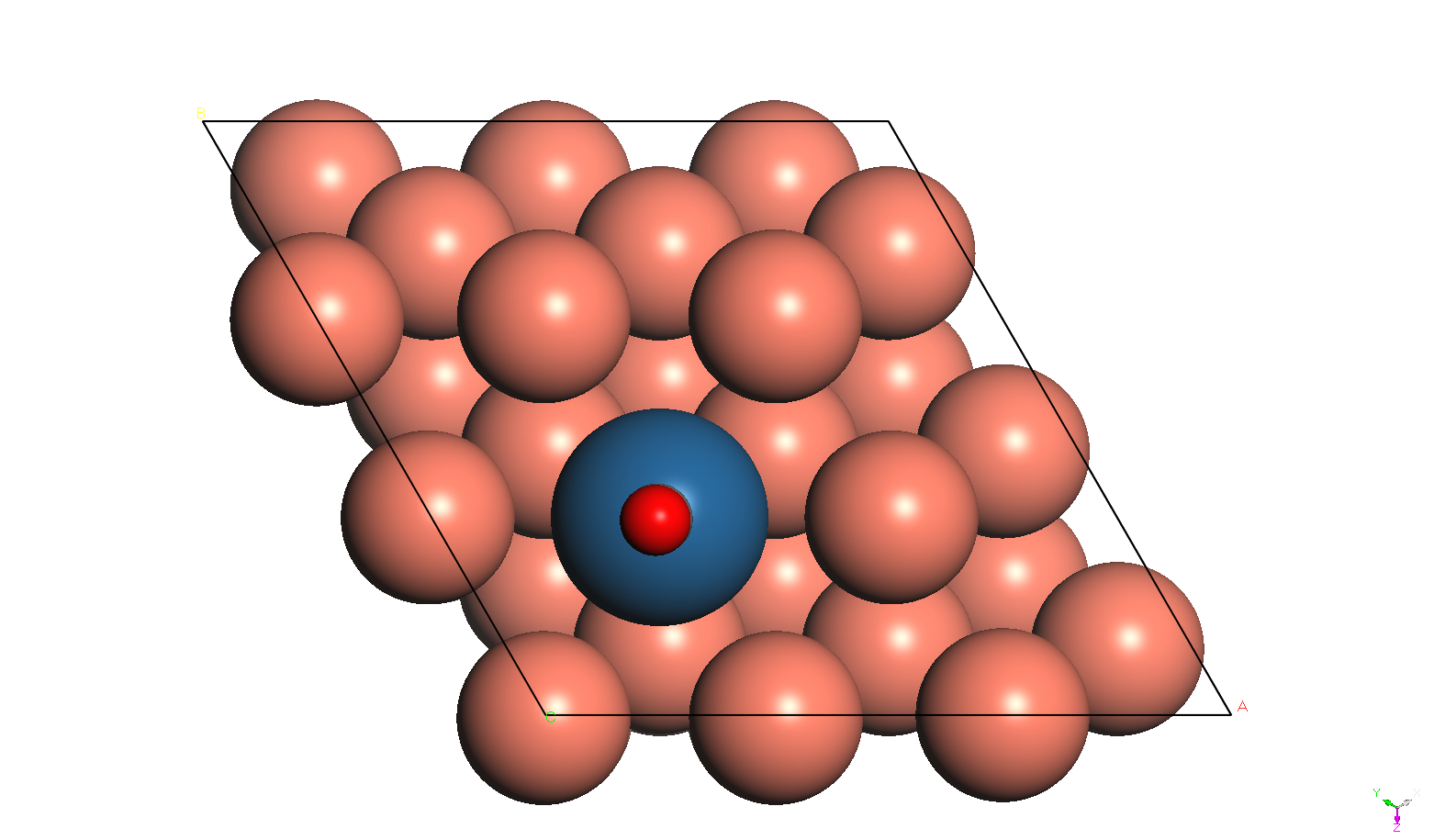

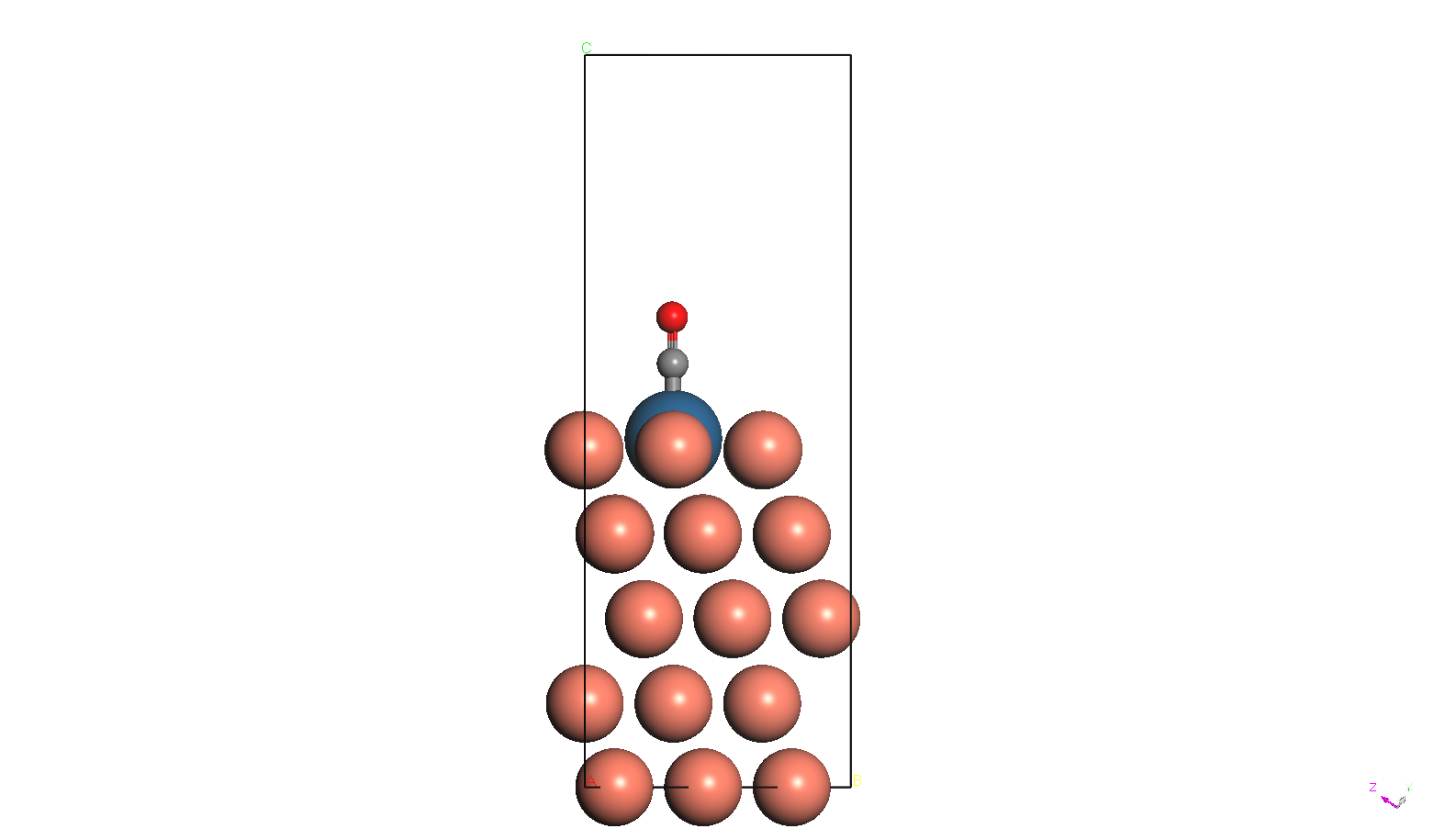


Figure 1: DFT optimized adsorption configuration for CO on a Pt/Cu(111) SAA from a top-down (left) and side-on (right) perspective. This configuration is the most stable for CO adsorption Pt/Cu(111) SAA and is analogous to on other SAA surfaces. For all SAAs, attempting to relax CO in a shared hollow or bridge site results in CO displacement back to this configuration.


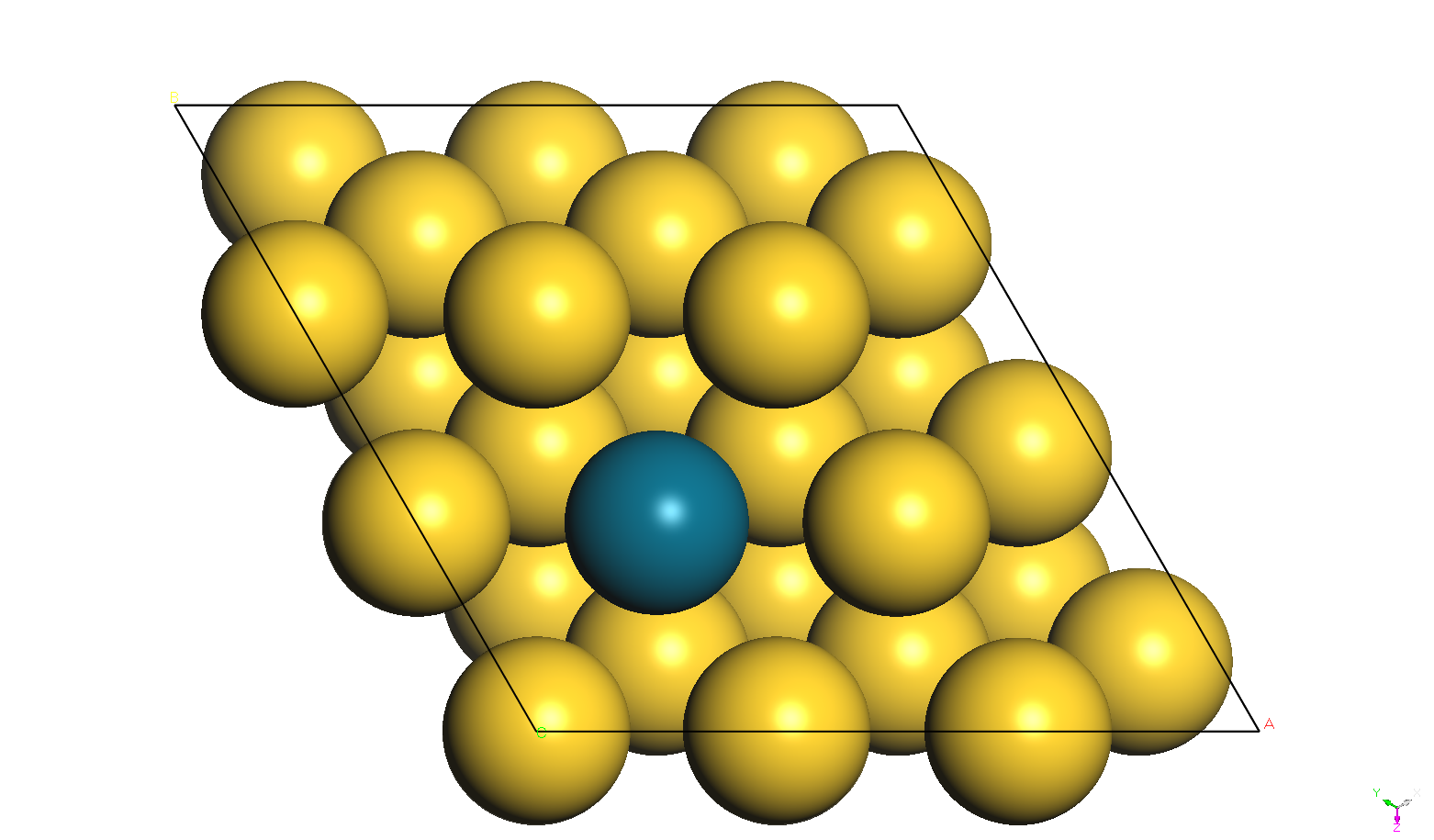

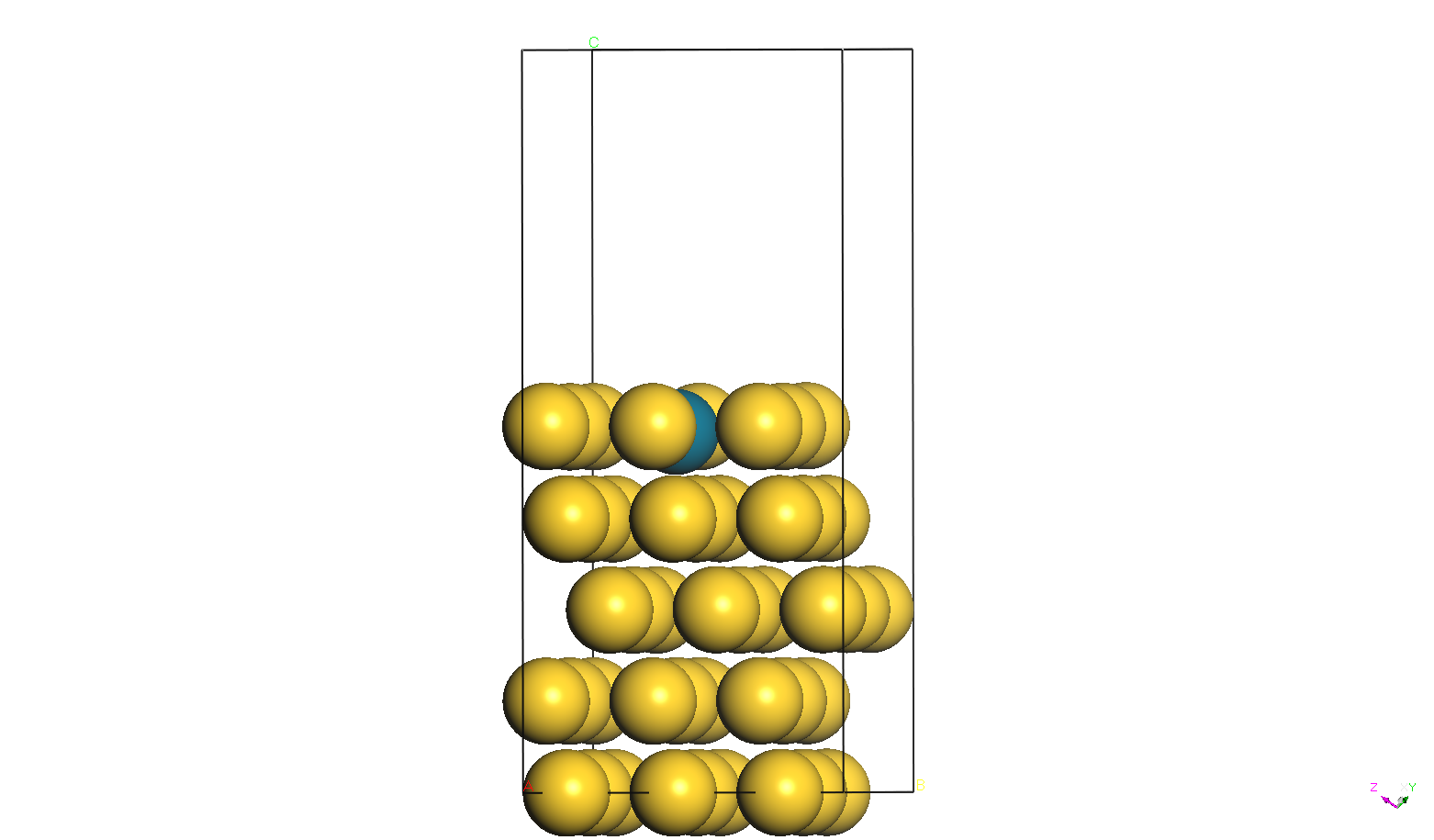

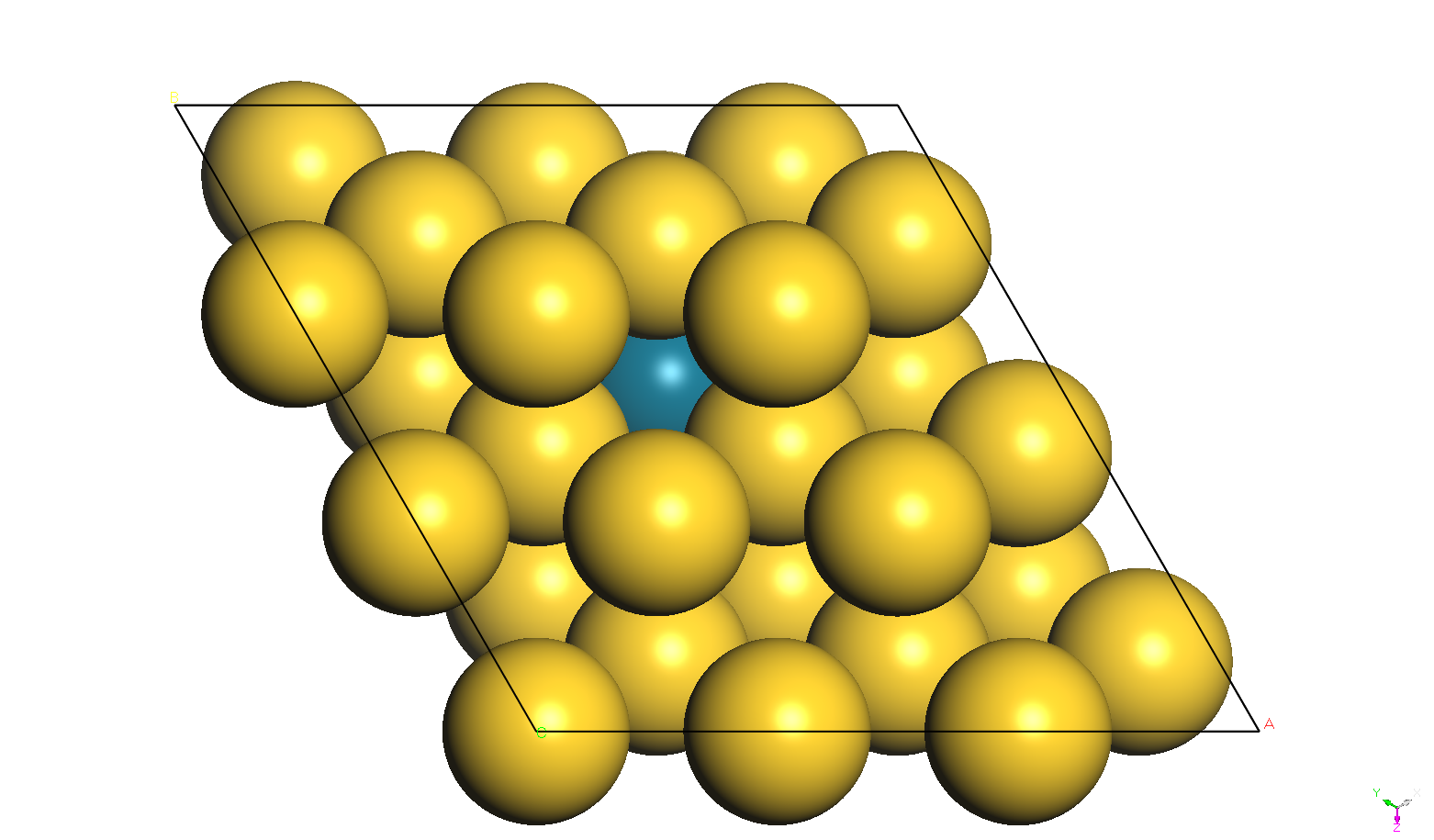

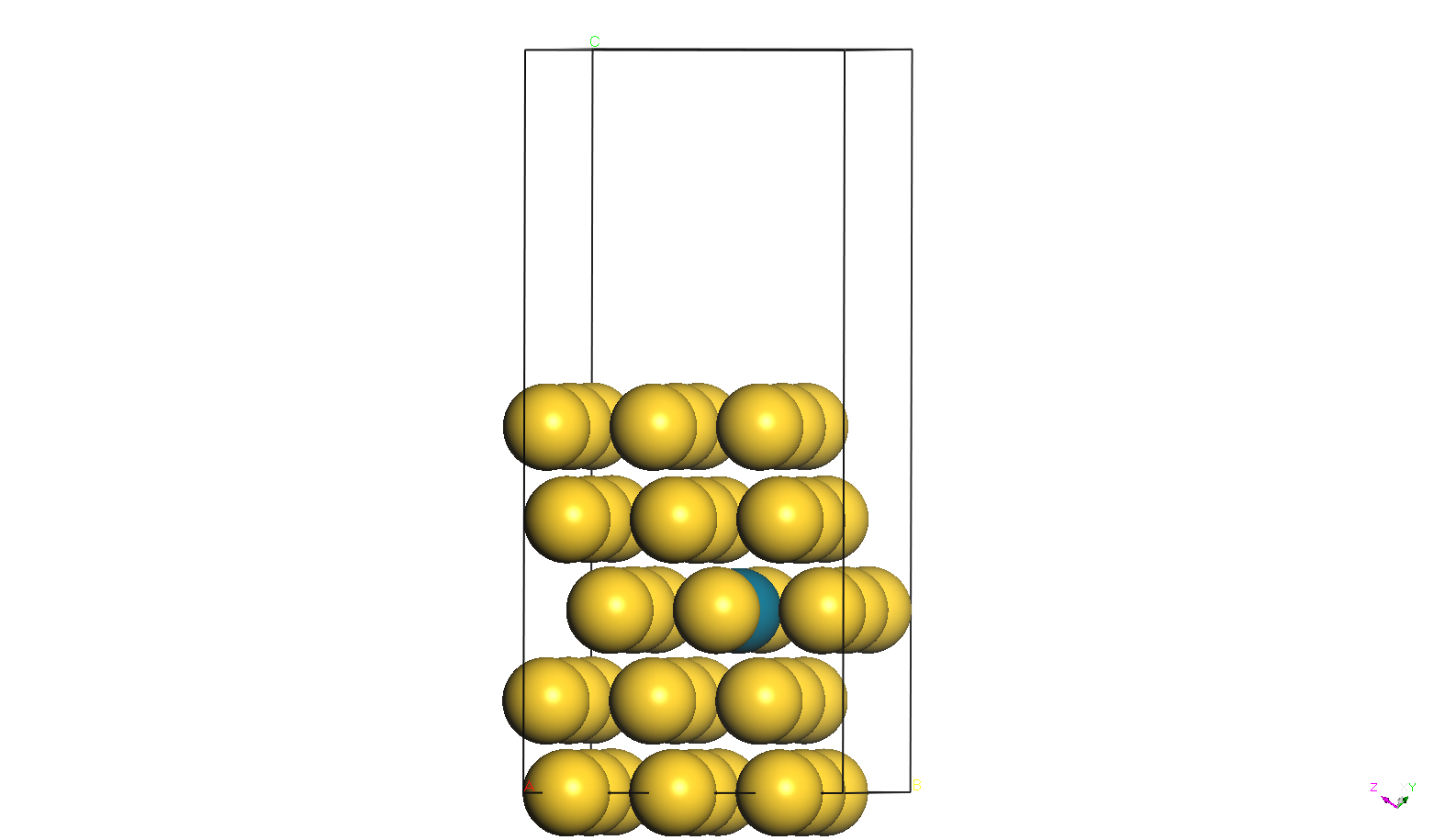


SAA

“Bulk”

Impurity

ΔE_seg_ > 0

ΔE_seg_ < 0

Segregation

Dopant Atom

Figure 2: Diagram illustrating the dopant atom segregation to/from the SAA phase (top) from/to the “bulk” (bottom). Negative values of the segregation energy (ΔE_seg_) imply an enthaplic preference for the single atom to be submerged in the bulk, whereas positive values indicate the single atom is more stable in the surface layer as a SAA.

$\Delta E_{seg}$

$\Delta E_{seg}^{CO}$

**SAA**

**“Bulk”**

**CO_(g)_**

**CO***

$-\Delta E_{seg}^{CO}$

$-\Delta E_{seg}$

$$E_{ads}^{host}(CO)$$

$$-E_{ads}^{host}(CO)$$

$$E_{ads}^{SAA}(CO)$$

$$-E_{ads}^{SAA}(CO)$$

**iv**

**ii**

**i**

**iii**

**= CO**

**= dopant**

**= host**

Figure 3: Energetic cycle for the segregation of a single dopant atom from the bulk to the surface layer of a host material in the presence and absence of CO. This cycle shows the link between the energies we define in the manuscript for the CO adsorption ($\boldsymbol{E}_{\boldsymbol{ads}}\boldsymbol{(CO)}$), the segregation energy ($\boldsymbol{\Delta}\boldsymbol{E}_{\boldsymbol{seg}}$) and the CO induced segregation energy ($\boldsymbol{\Delta}\boldsymbol{E}_{\boldsymbol{seg}}^{\boldsymbol{CO}}$). Conversions denoted by Roman numerals correspond to those discussed in the main manuscript, section “CO Induced Surface Segregation”.


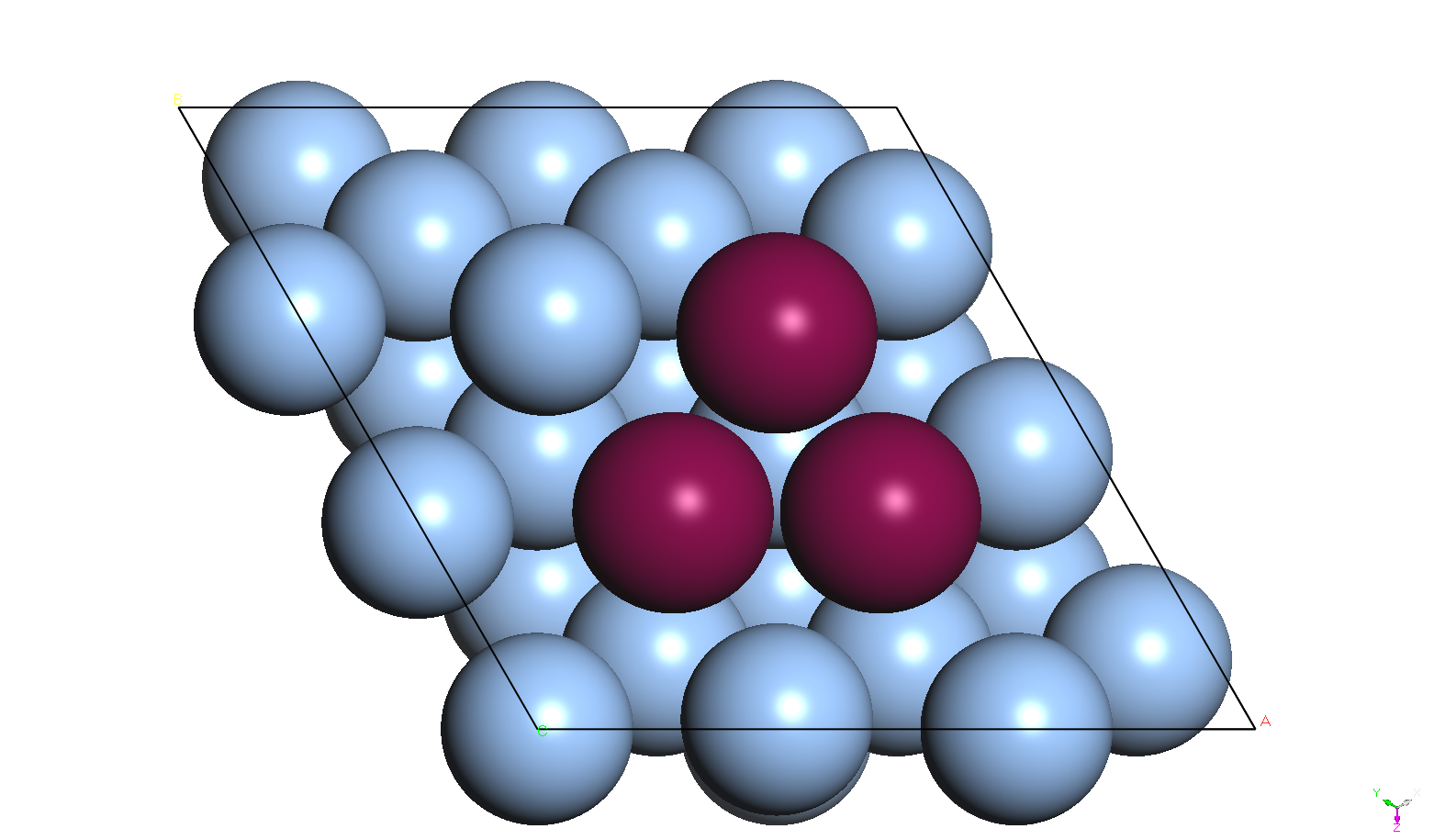

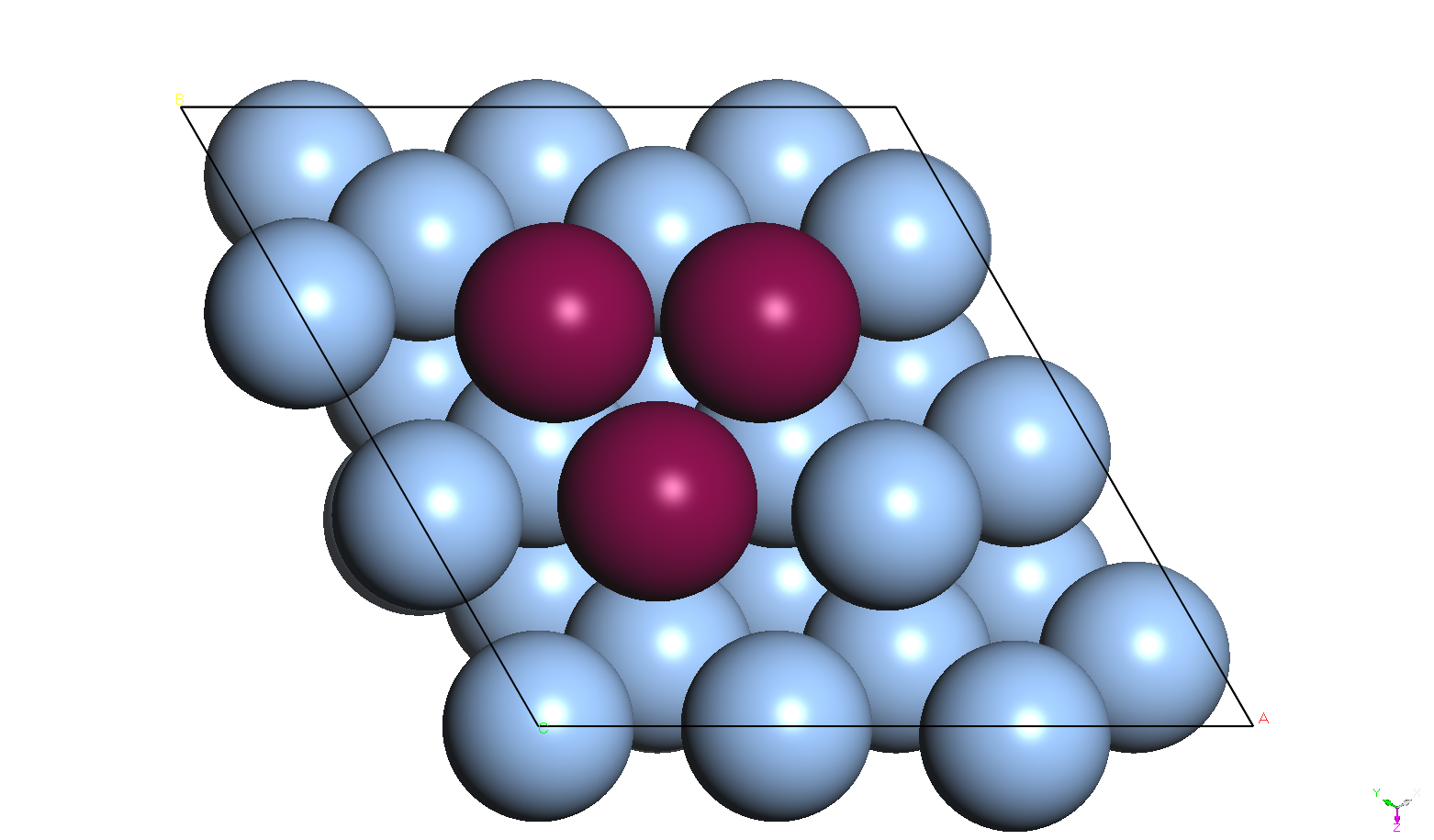

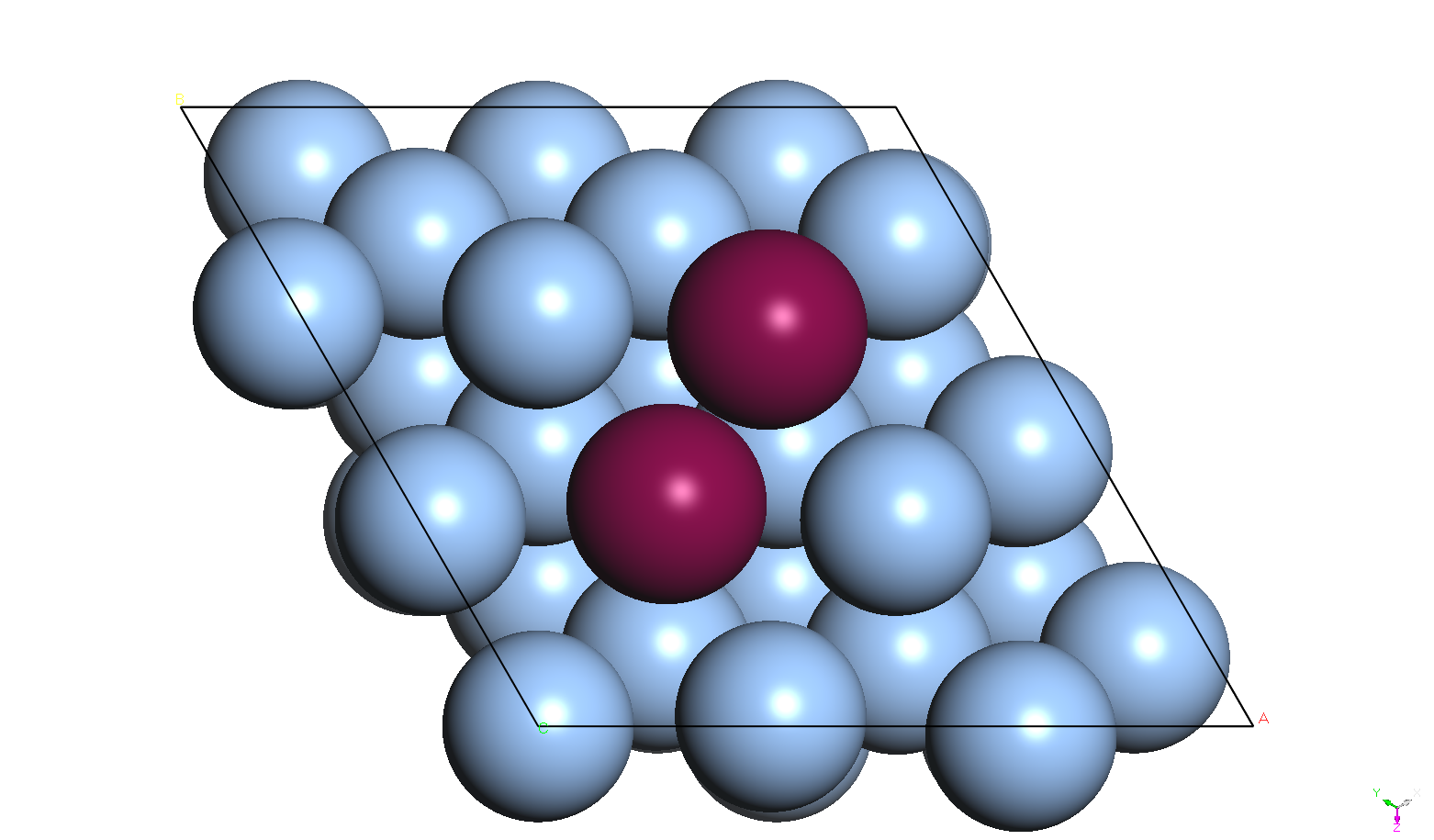

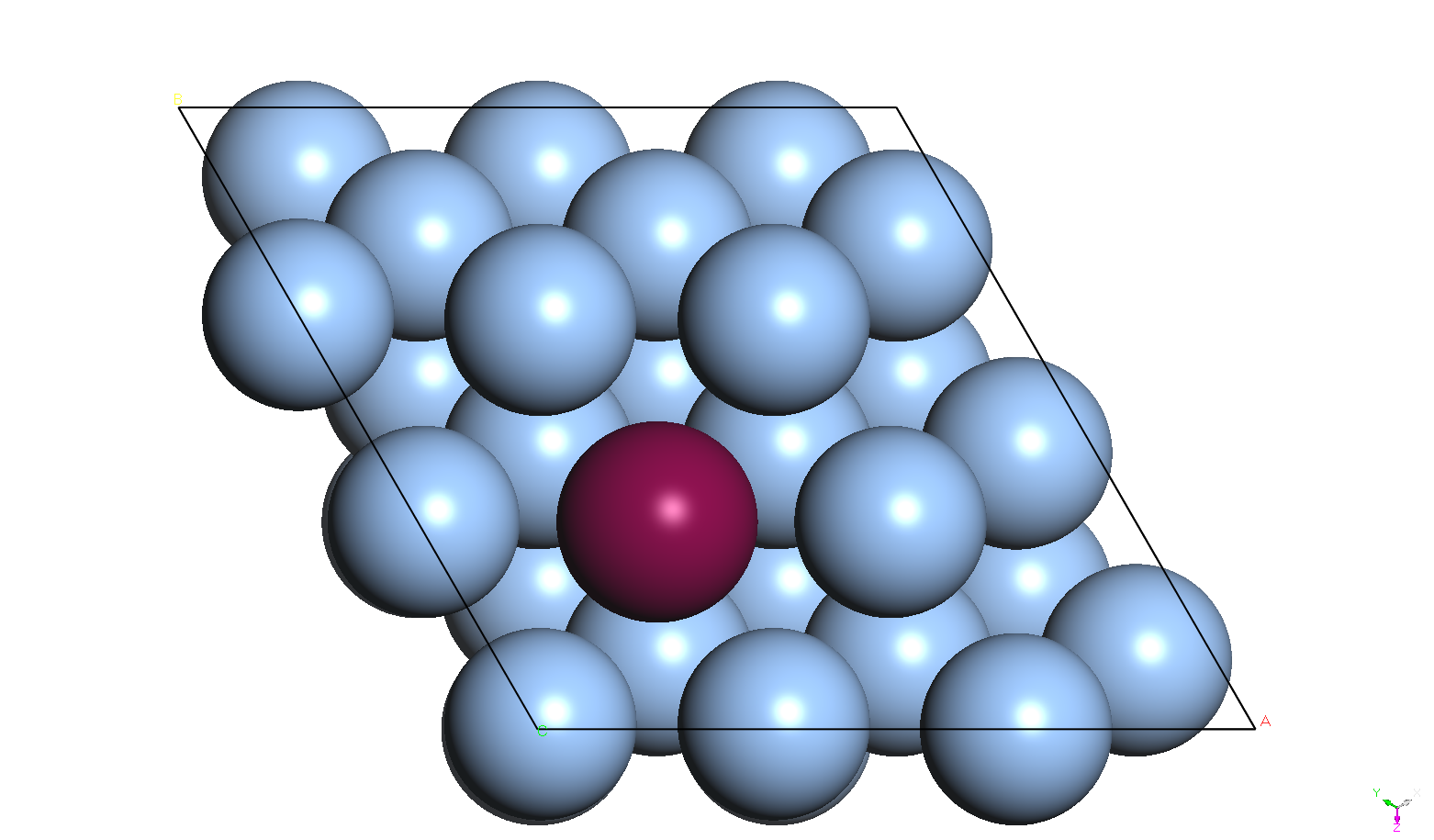


SAA

Trimer (hcp)

Trimer (fcc)

Dimer

Figure 4: DFT optimized monomer (SAA), dimer and trimer (surrounding hcp and fcc sites) configurations for binary alloys of Ir-doped Ag(111) surfaces. This metal combination has negative values of ΔE_agg­_ indicating a preference for clustering of dopant metal atoms rather than dispersion. Other alloys with ΔE_agg­_ < 0 have comparable structures to these.


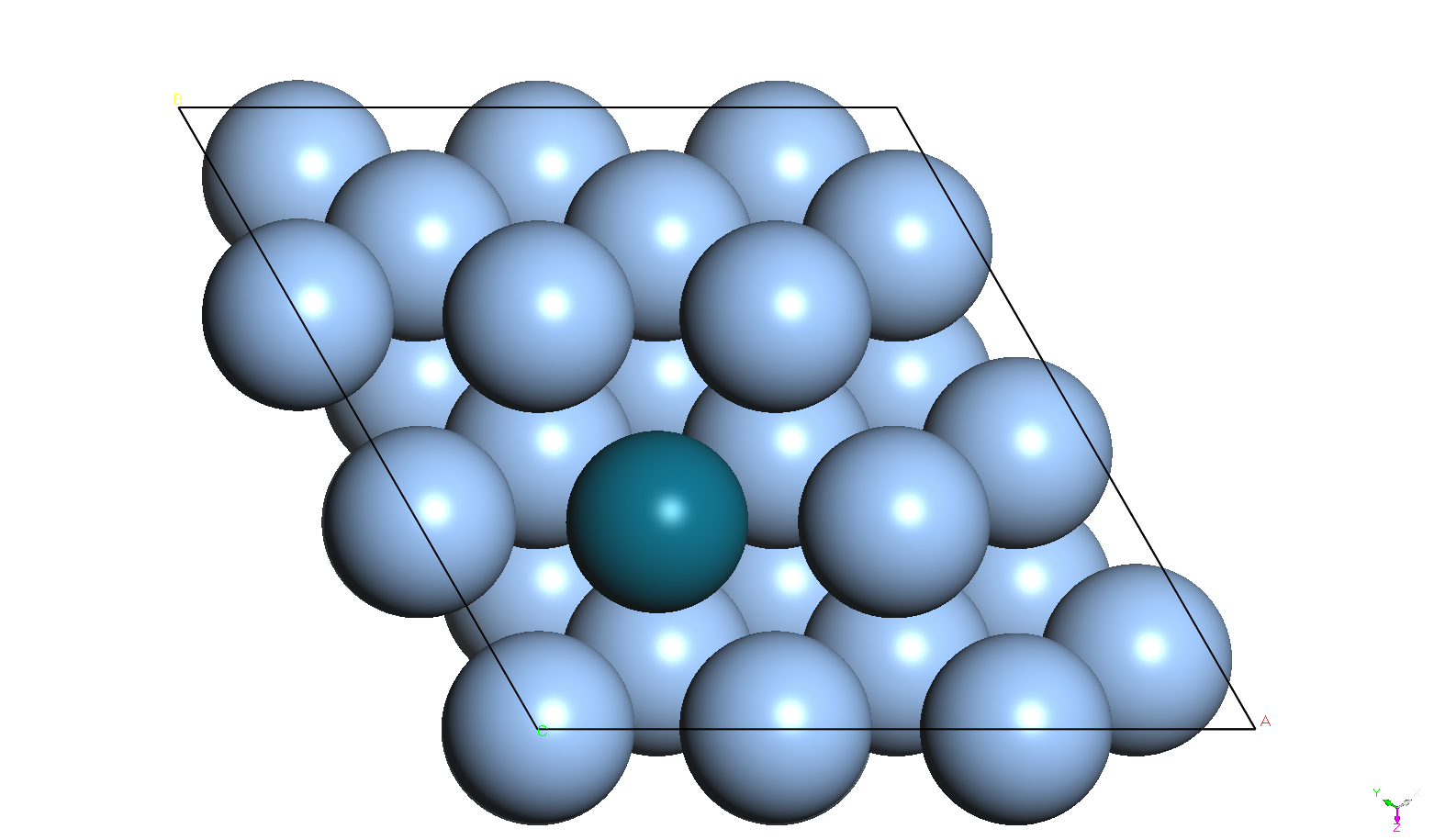

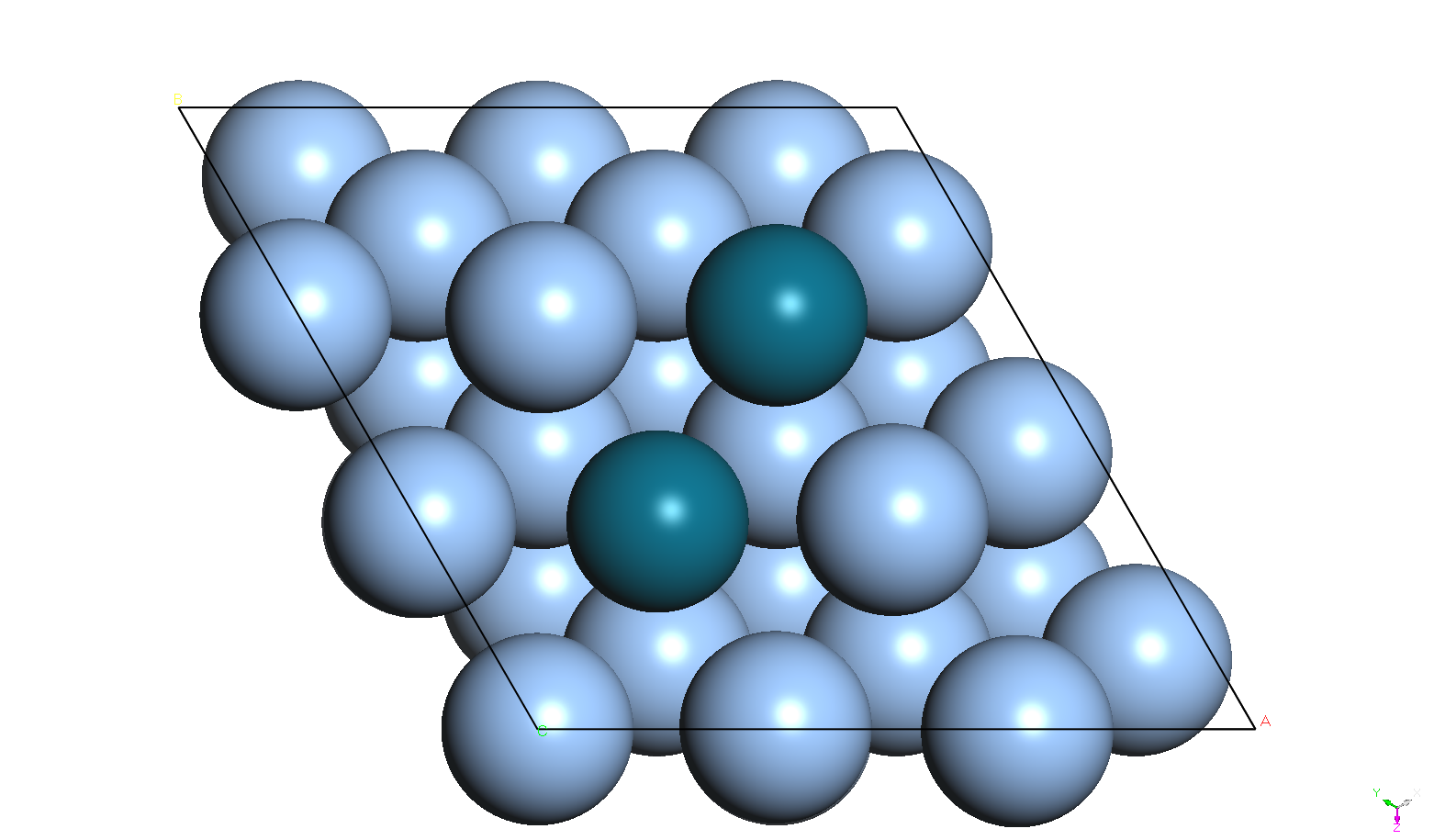

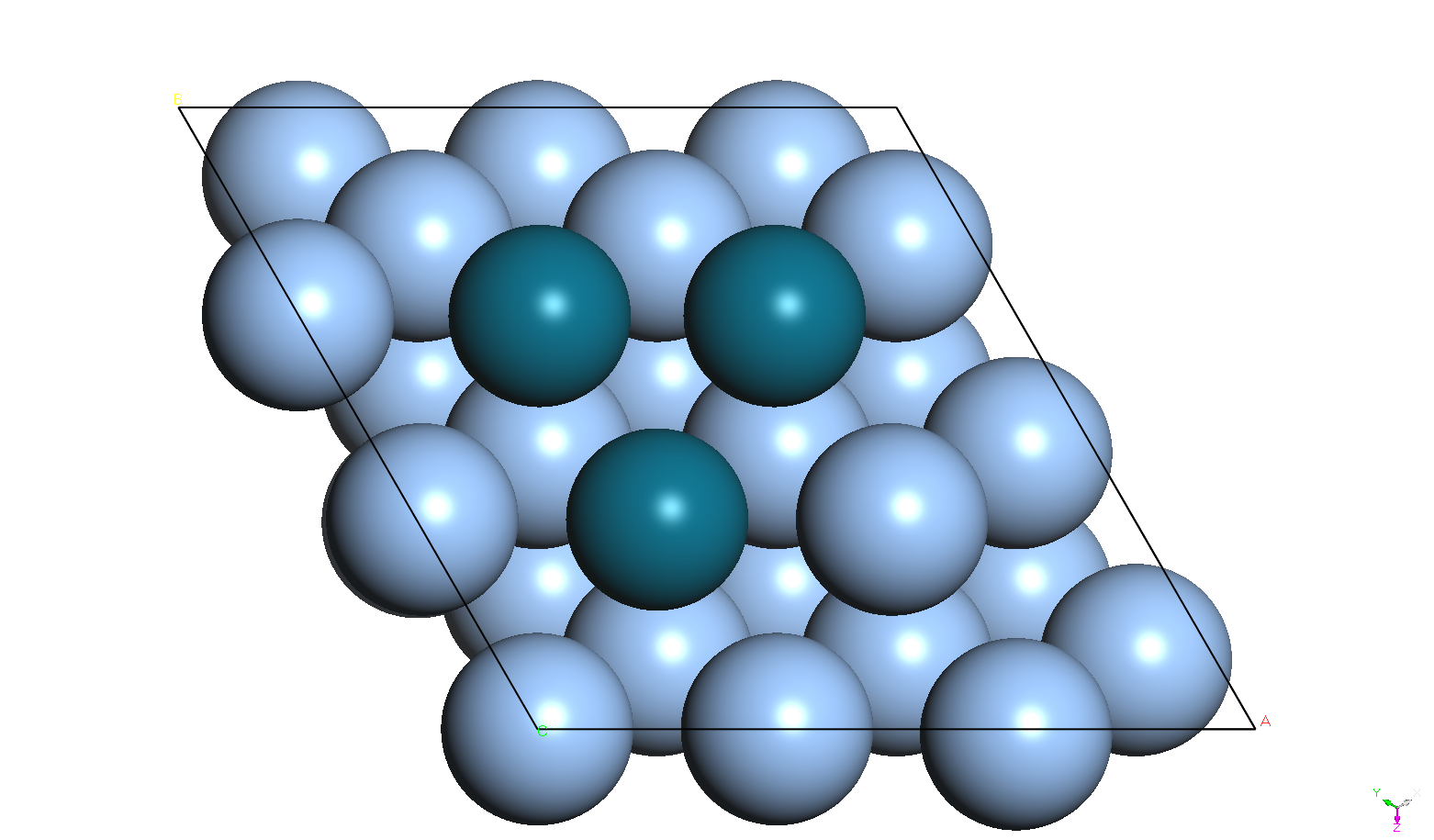

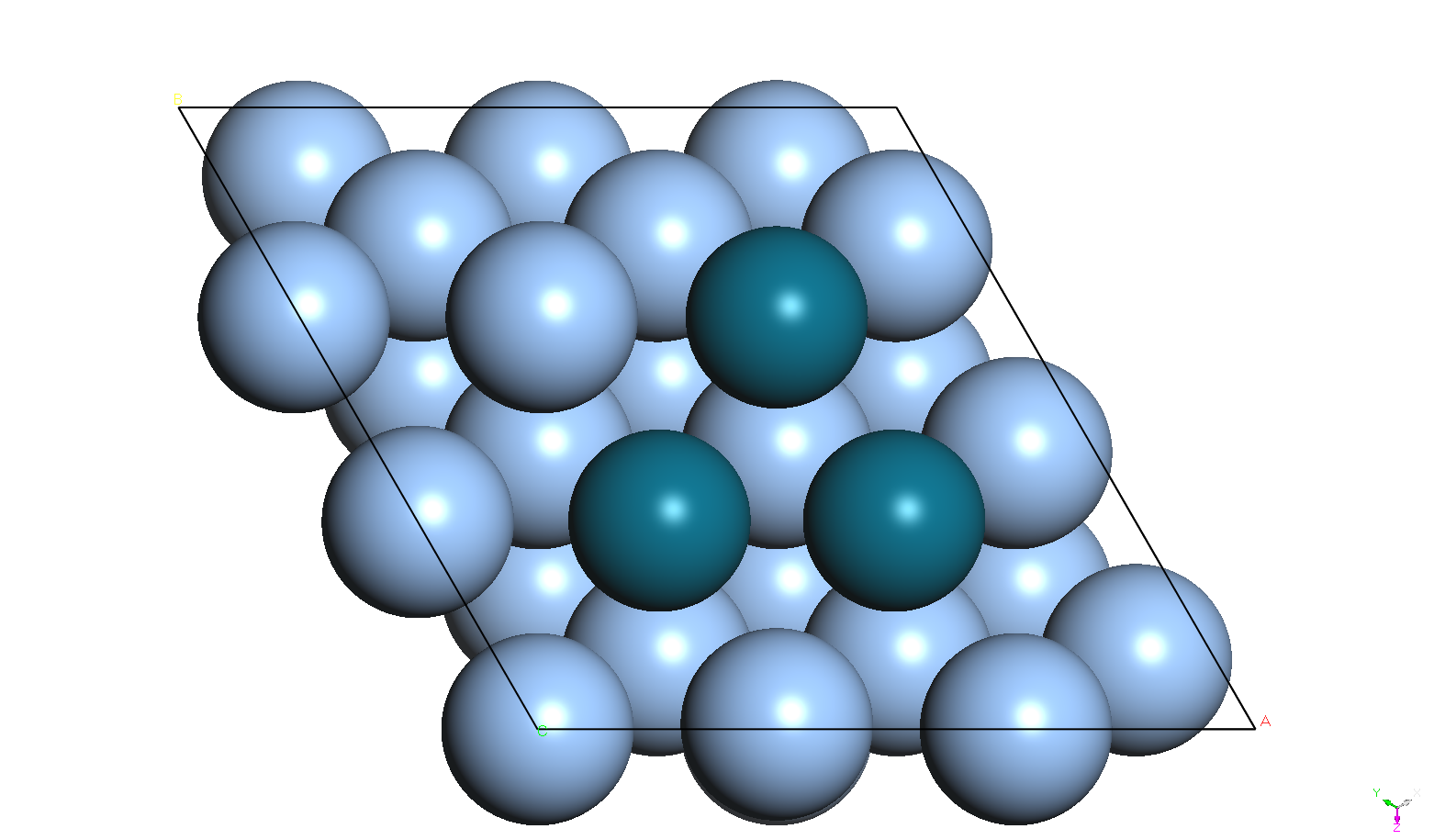


SAA

Dimer

Trimer (fcc)

Trimer (hcp)

Figure 5: DFT optimized monomer (SAA), dimer and trimer (surrounding hcp and fcc sites) configurations for binary alloys of Pd-doped Ag(111) surfaces. This metal combination has positive values of ΔE_agg­_ indicating a preference for dispersion of surface dopant atoms rather than clustering. Other alloys with ΔE_agg­_ > 0 have comparable structures to these.


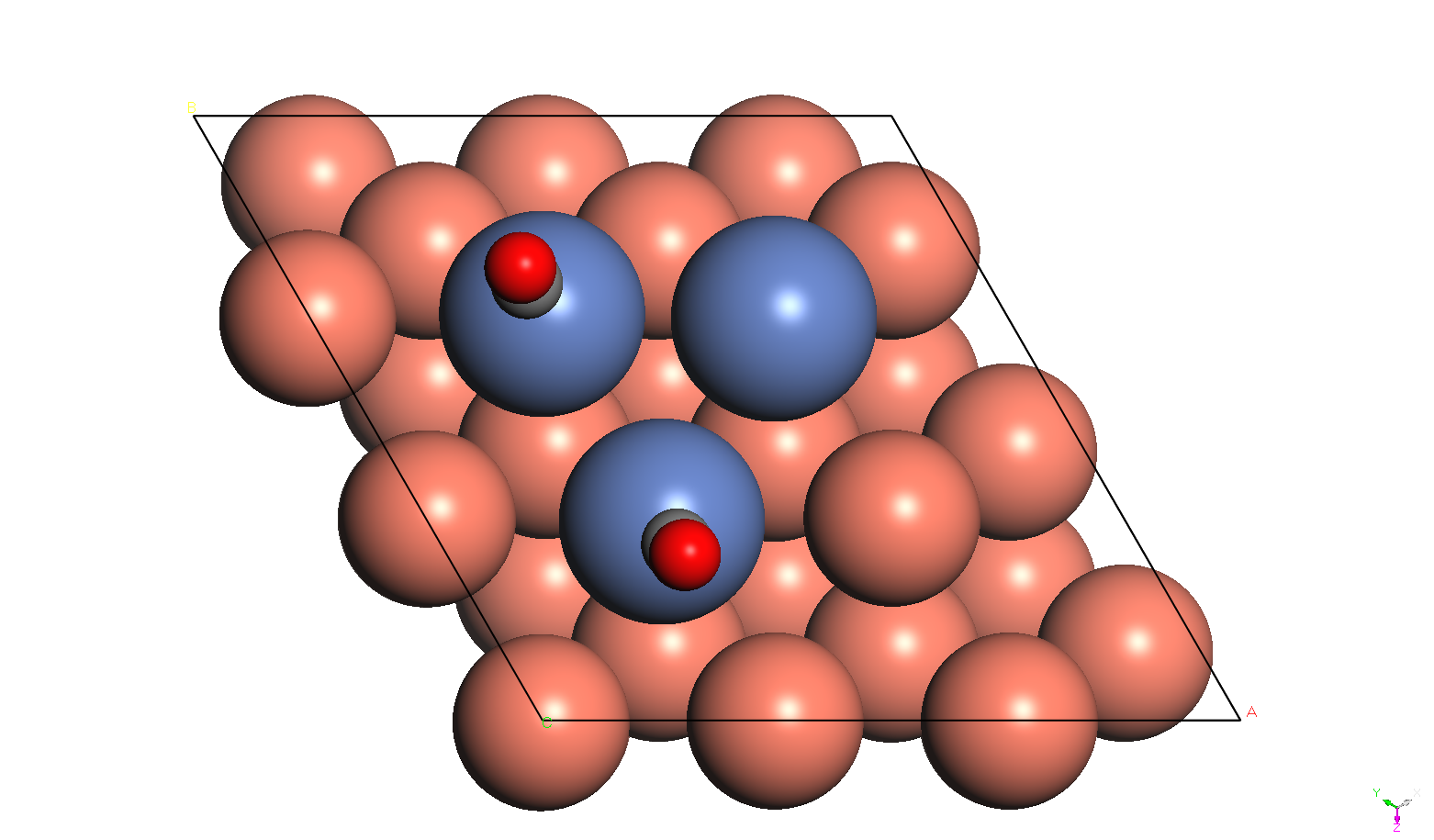

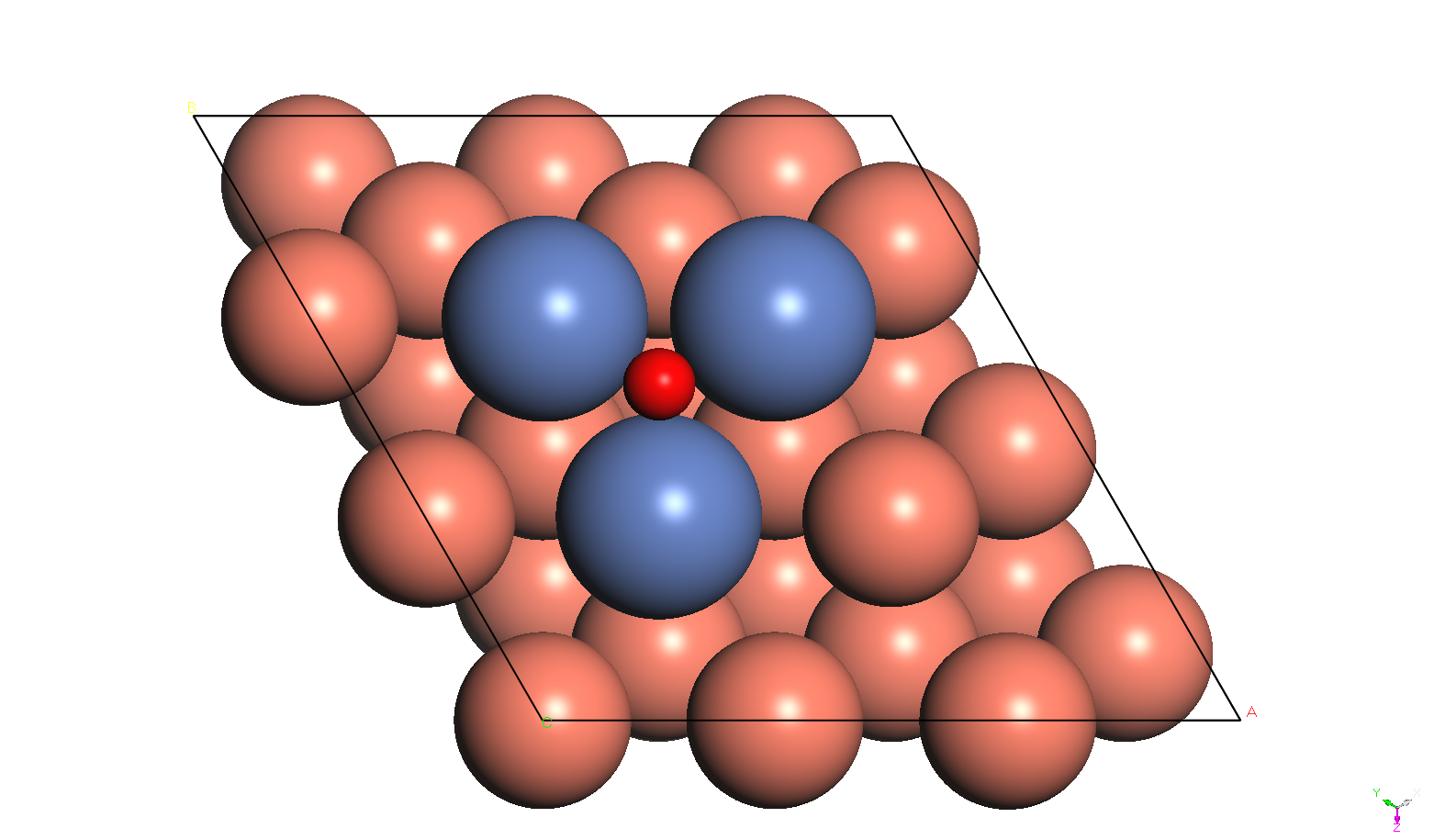

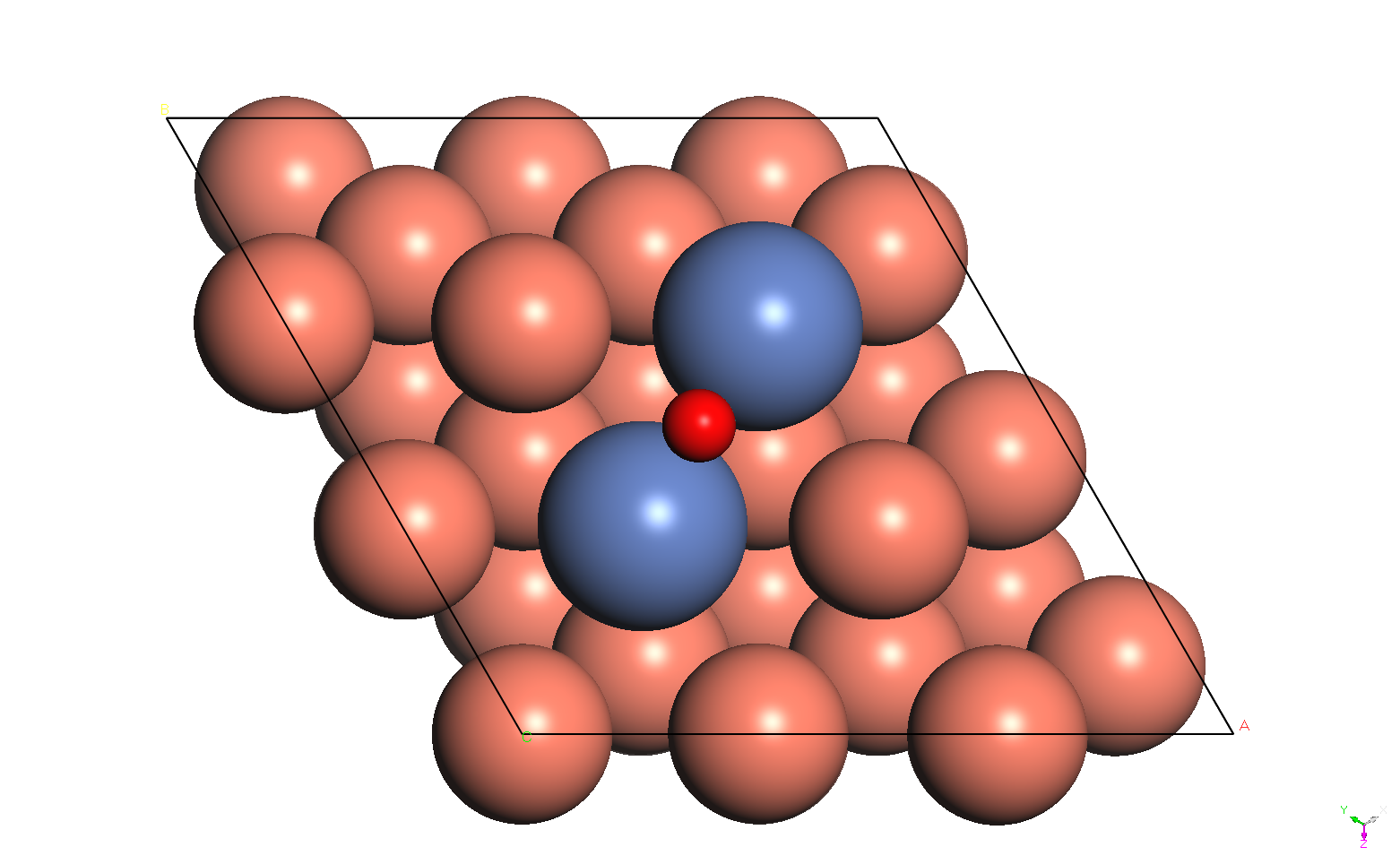

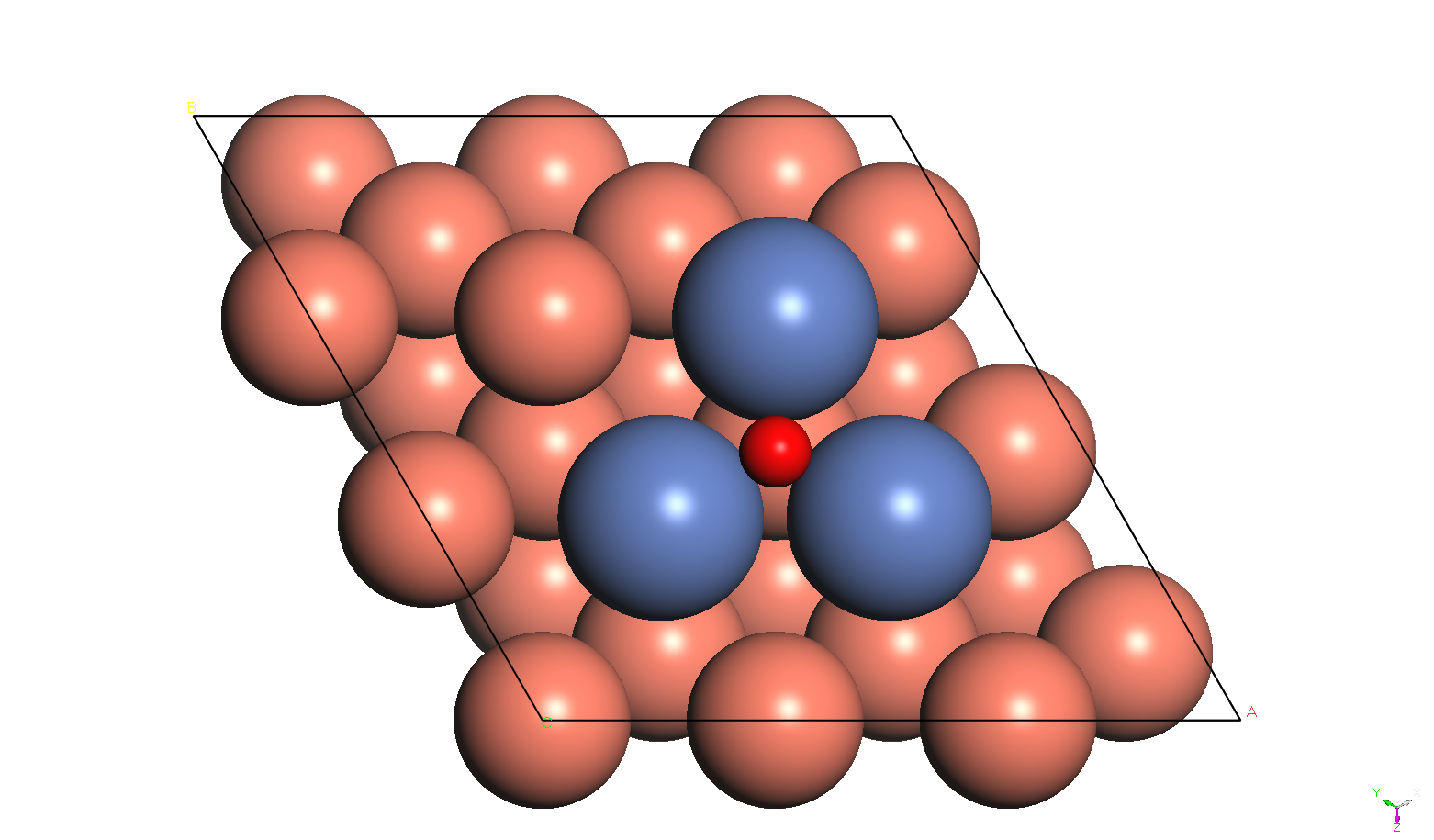

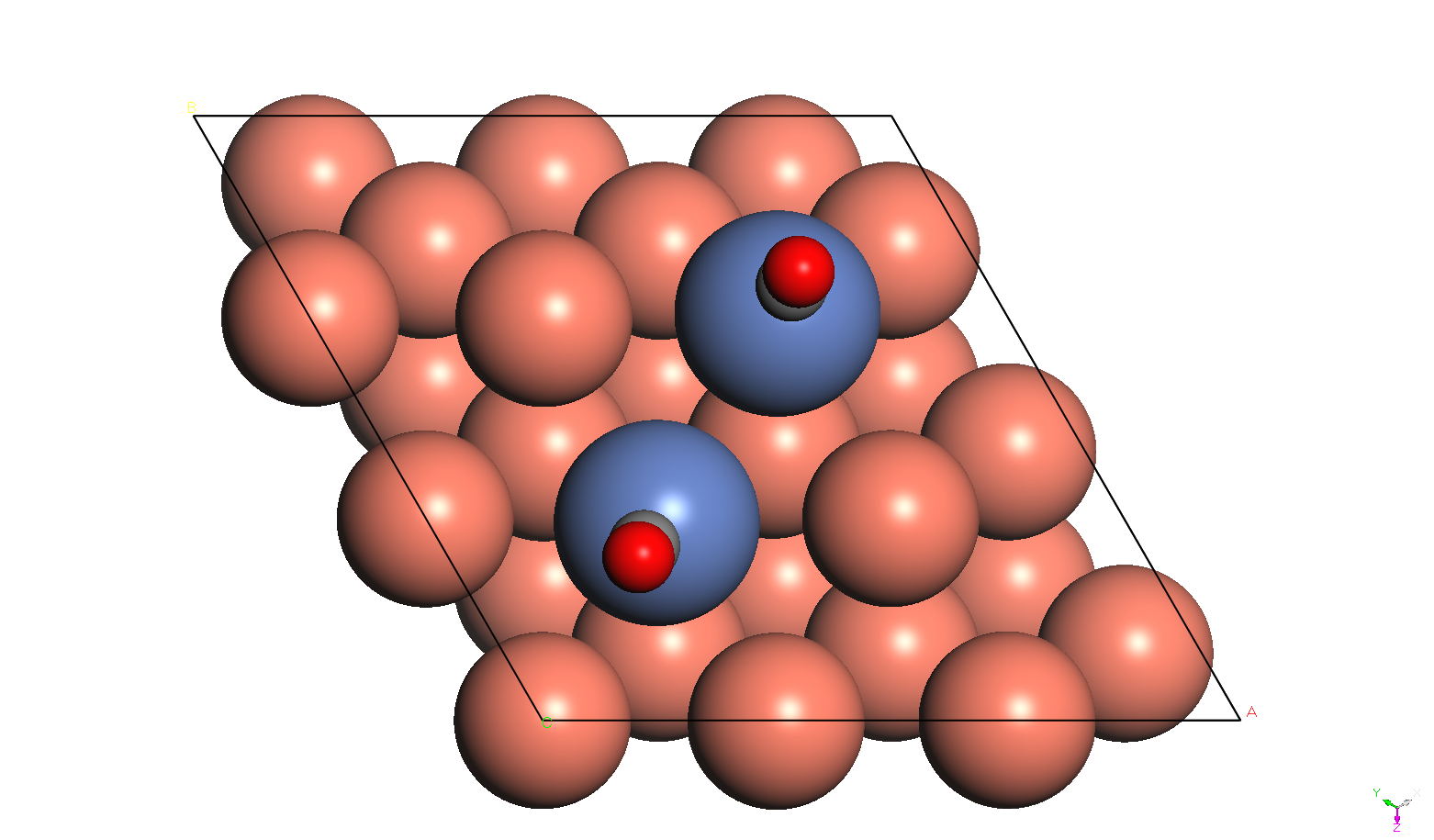

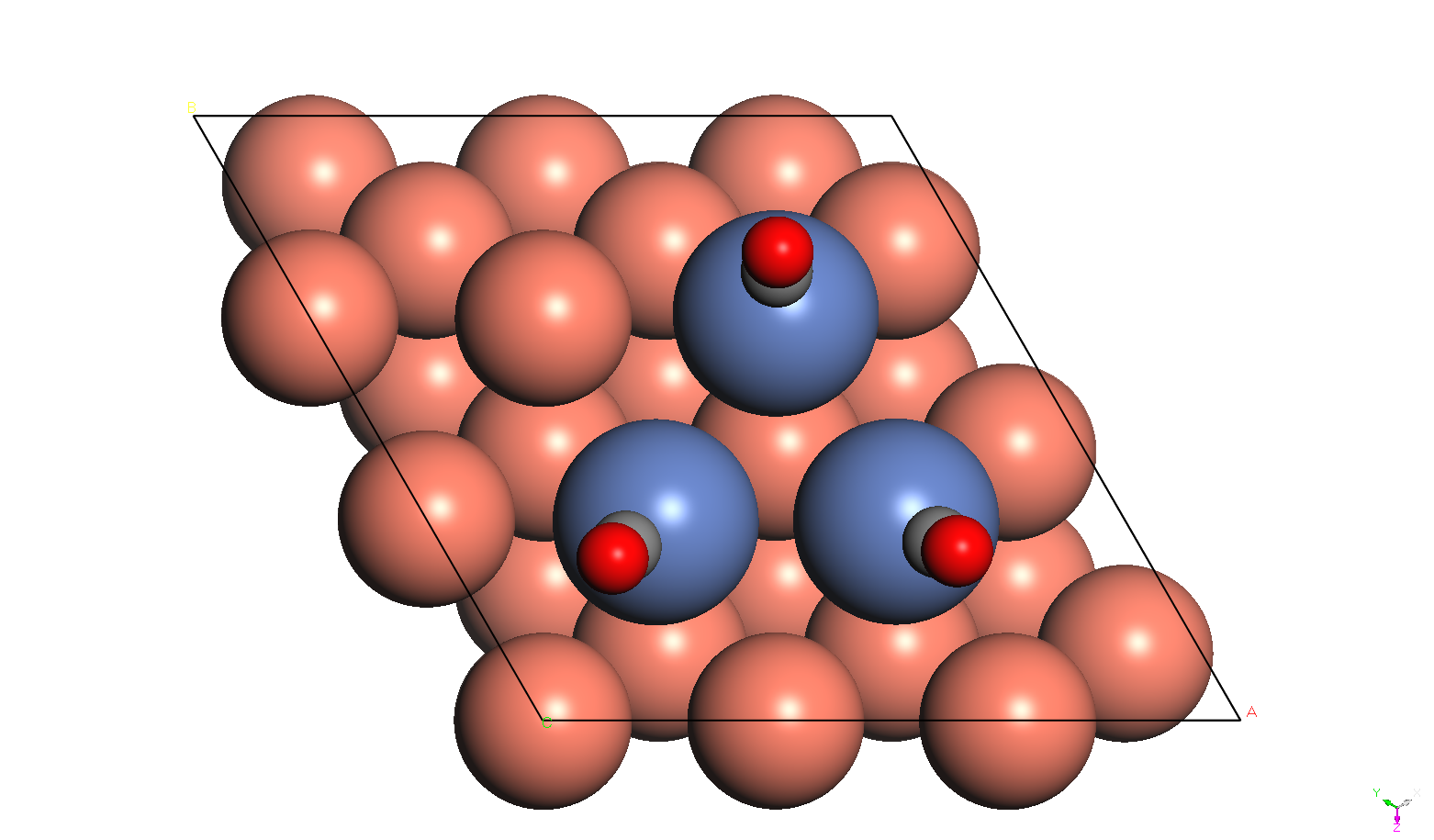

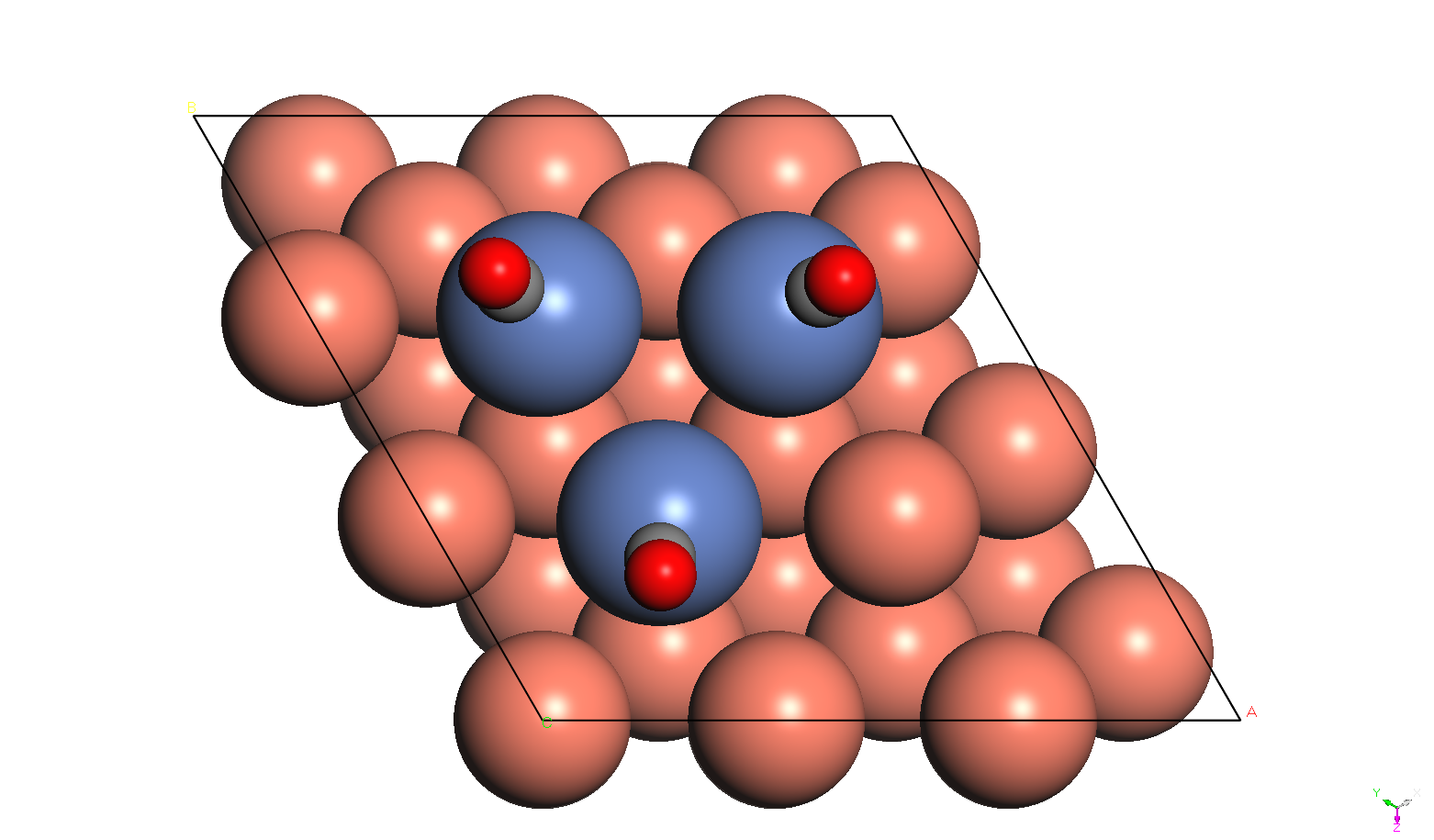

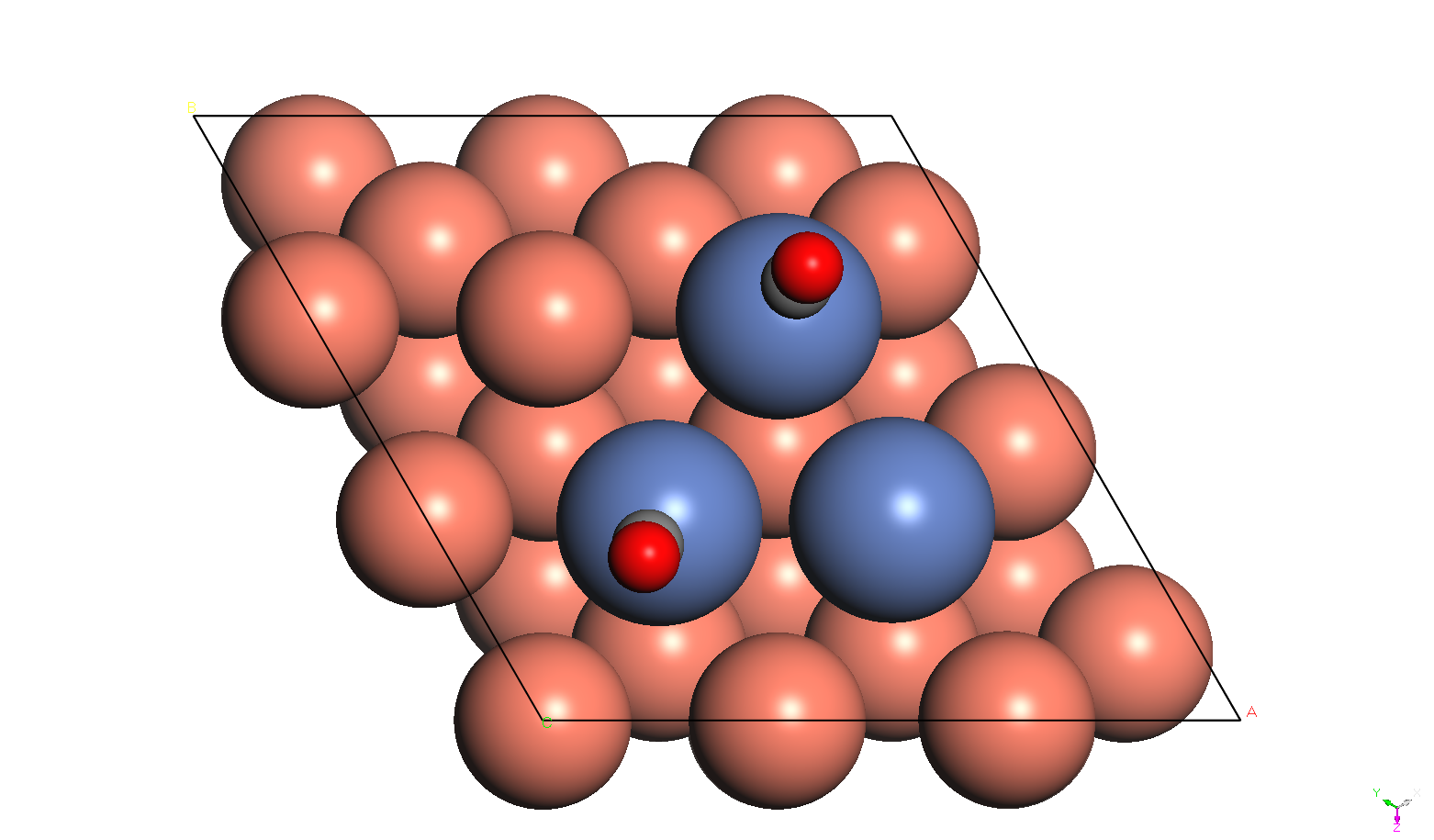


1 x CO Molecule

2 x CO Molecules

3 x CO Molecules

Figure 6: DFT optimized structures for 1, 2 and 3 CO molecules (co-)adsorbed on Ni-doped Cu(111) binary alloy dimers and trimers. These are the most stable adsorption structures for the given coverage. For 1 x CO molecule, these most favoured structures are comparable to those on all other surfaces in this study except for Pt/Cu(111), Ir/Ag(111), Ir/Au(111) and Ir/Cu(111) whereby top site CO adsorption is preferred on dimers and trimers. For 2 and 3 CO molecules, these structures are comparable to the most favoured structures on all other surfaces.


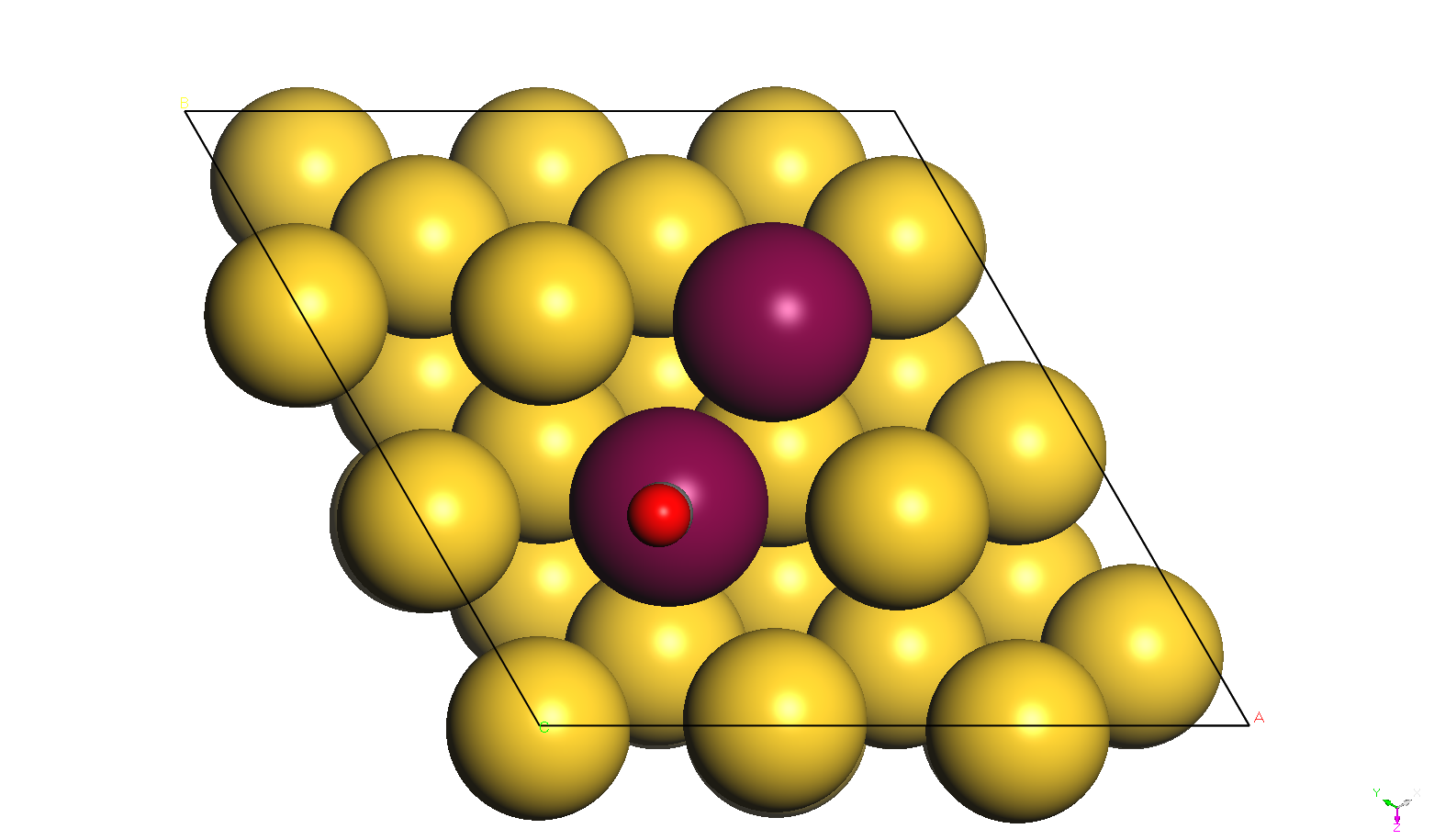

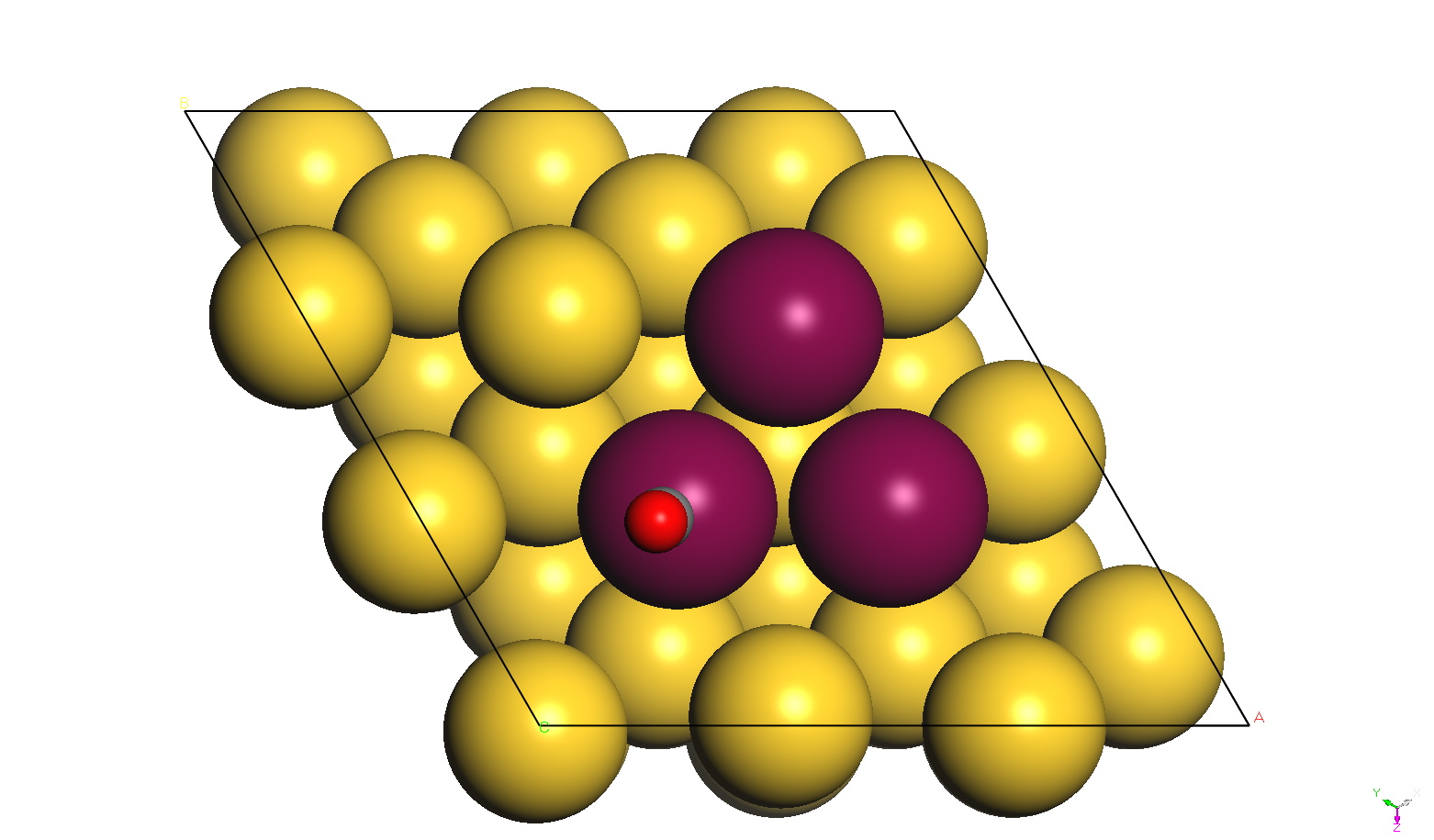

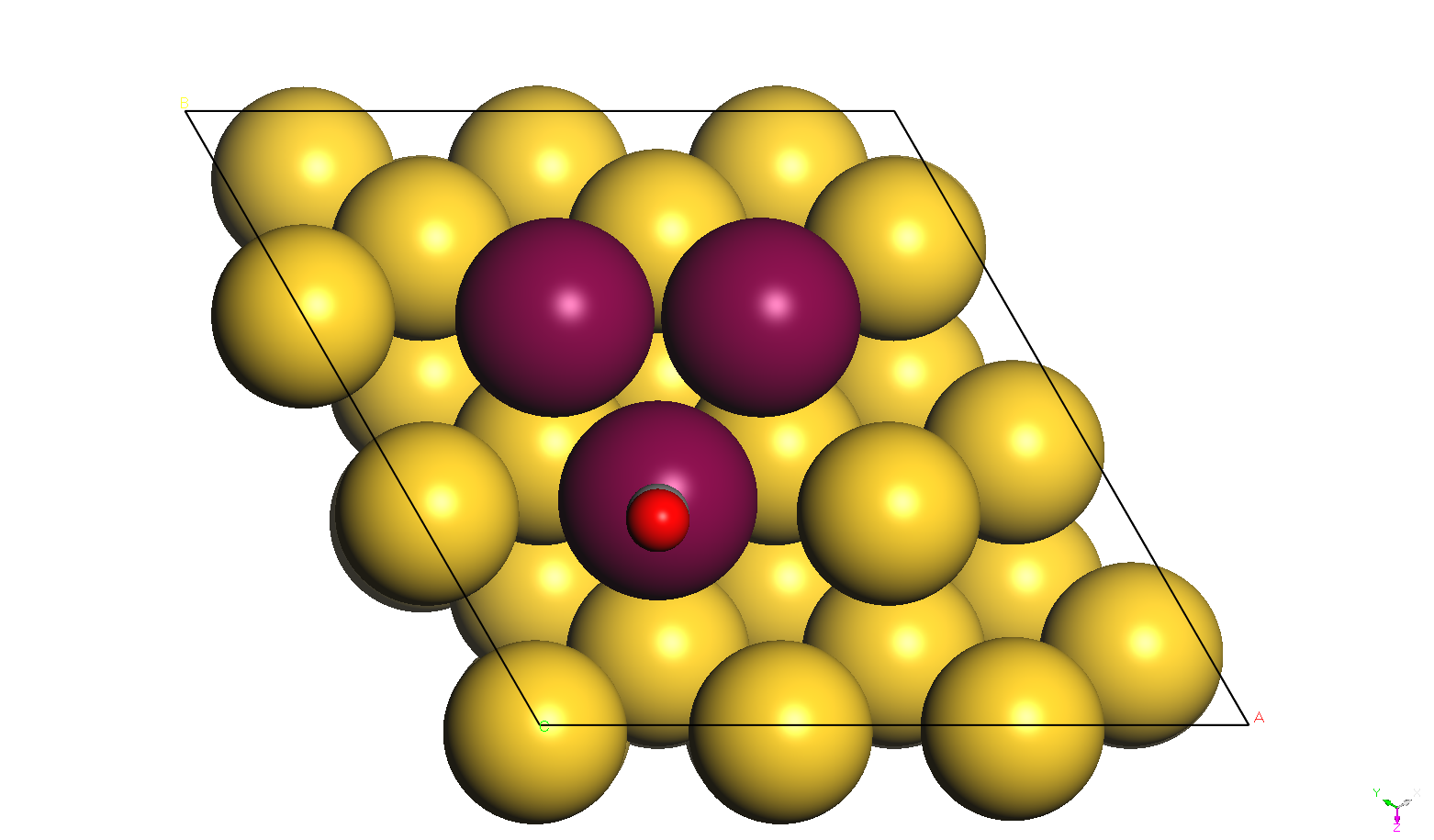


Figure 7: DFT optimized structures for 1 CO molecule adsorbed on Ir-doped Au(111) binary alloy dimers and trimers. These are the most stable adsorption structures for the given coverage. For 1 x CO molecule, these most favoured structures are comparable to those on Pt/Cu(111), Ir/Ag(111), and Ir/Cu(111).
